# Ex vivo *e*xpanded allogeneic natural killer cells have potent cytolytic activity against cancer cells through different receptor-ligand interactions

**DOI:** 10.1186/s13046-021-02089-0

**Published:** 2021-10-23

**Authors:** Daun Jung, Young Seok Baek, In Jee Lee, Ki Yeon Kim, Heejoo Jang, Sohyun Hwang, Jieun Jung, Yong-wha Moon, Kyung-Soon Park, Yong-Soo Choi, Hee Jung An

**Affiliations:** 1 Department of Pathology, CHA Bundang Medical Center, CHA University, 59 Yatapro Sungnam, Gyeonggi-do, Seongnam, Republic of Korea; 2 Immunotherapy Team, New Biological Entity (NBE) Research, R&D Division, CHA Biotech, Seongnam, Republic of Korea; 3 Department of Biomedical Science, CHA University, Seongnam, Republic of Korea; 4 Center for Research & Development, CHA Advanced Research Institute, Seongnam, Republic of Korea; 5 Department of Medical Oncology, CHA Bundang Medical Center, CHA University, Seongnam, Republic of Korea

**Keywords:** Natural killer cells, NK-activating receptors, Inhibitory receptors, Allogeneic, Cryopreservation, cancer immunotherapy

## Abstract

**Background:**

Recently, allogeneic natural killer (NK) cells have gained considerable attention as promising immunotherapeutic tools due to their unique biological functions and characteristics. Although many NK expansion strategies have been reported previously, a deeper understanding of cryopreserved allogeneic NK cells is needed for specific therapeutic approaches.

**Methods:**

We isolated CD3^−^CD56^+^ primary natural killer (pNK) cells from healthy donors and expanded them ex vivo using a GMP-compliant method without any feeder to generate large volumes of therapeutic pNK cells and cryopreserved stocks. After validation for high purity and activating phenotypes, we performed RNA sequencing of the expanded and cryopreserved pNK cells. The pNK cells were used against various cancer cell lines in 7-AAD/CFSE cytotoxicity assay. For in vivo efficacy study, NSG mice bearing subcutaneous cisplatin-resistant A2780cis xenografts were treated with our pNK cells or cisplatin. Antitumor efficacy was assessed by measuring tumor volume and weight.

**Results:**

Compared to the pNK cells before expansion, pNK cells after expansion showed 2855 upregulated genes, including genes related to NK cell activation, cytotoxicity, chemokines, anti-apoptosis, and proliferation. Additionally, the pNK cells showed potent cytolytic activity against various cancer cell lines. Interestingly, our activated pNK cells showed a marked increase in NKp44 (1064-fold), CD40L (12,018-fold), and CCR5 (49-fold), and did not express the programmed cell death protein 1(PD-1). We also demonstrated the in vitro and in vivo efficacies of pNK cells against cisplatin-resistant A2780cis ovarian cancer cells having a high programmed death-ligand 1(PD-L1) and low HLA-C expression.

**Conclusions:**

Taken together, our study provides the first comprehensive genome wide analysis of ex vivo-expanded cryopreserved pNK cells. It also indicates the potential use of expanded and cryopreserved pNK cells as a highly promising immunotherapy for anti-cancer drug resistant patients.

**Supplementary Information:**

The online version contains supplementary material available at 10.1186/s13046-021-02089-0.

## Background

Natural killer (NK) cells belong to the innate lymphoid cell family, and they account for approximately 5–15% of peripheral blood lymphocytes [1, 2]. In humans, NK cells are phenotypically characterized by the absence of surface T-cell receptors and associated CD3 molecules and expression of neural cell adhesion molecule, also known as CD56 [3]. As the name suggests, NK cells possess “natural” ability to kill tumor and virus-infected cells without any priming or prior sensitization in contrast to cytotoxic T cells [4].

NK cells directly kill target cells through apoptosis by releasing cytoplasmic granules containing perforin, granzymes, and death receptors [5, 6]. NK cells also modulate other immune cells by releasing several cytokines, such as interferon (IFN)-ɣ and tumor necrosis factor (TNF)-ɑ, and this is followed by ligand interaction with cell-surface receptors [7]. NK cell activity is controlled by signals derived from a variety of activating and inhibitory receptors expressed on the cell surface [6, 8]. The NK cell activating signal is mediated by several NK receptors, including natural killer group 2D (NKG2D), natural cytotoxicity receptors (NCRs; NKp46, NKp44, and NKp30), Ig-like receptors (2B4), and costimulatory receptors (DNAM-1). In contrast, NK cell inhibition is regulated by two major histocompatibility complex class I (MHC-1; also known as human leukocyte antigen (HLA))-specific inhibitory receptors, killer cell immunoglobulin-like receptors (KIRs) and the CD94-NKG2A heterodimer. Normal healthy cells express MHC class I molecules on their surface; therefore, they are protected from NK cell-mediated lysis. However, virus-infected or tumor cells lose surface MHC class I expression, leading to a low inhibitory NK cell signaling and allowing NK cells to become activated to kill target cells [2, 9].

Due to the advances in understanding NK cell receptors and their ligands, NK cell-based immunotherapy has emerged as a promising therapeutic approach for solid tumors and hematological malignancies. Allogeneic NK cell infusion is promising, especially for cancer immunotherapy, due to the “missing self” hypothesis that states that NK cells are able to sense the absence of “self” MHC class I on target cells [10] whereas autologous NK cell activity is inhibited in cancer patients largely due to KIR-HLA matching [11]. Unlike autologous NK cells, KIR-HLA mismatched allogeneic NK cells are expected to contribute to the killing of tumor cells that are resistant to autologous NK cells [12, 13]. Although a number of clinical trials are being conducted to evaluate the application of allogeneic NK-based immunotherapy [14, 15], more studies are needed to assess the best practices.

Peripheral blood mononuclear cells (PBMCs) have been used as one of the best sources of NK cells for clinical applications [16]. However, their clinical application is limited due to challenges in the production of a sufficient number of NK cells. Thus, ex vivo NK cell expansion is the most critical step in developing NK cell therapy. Various culture methods have been reported for ex vivo clinical grade-NK cell expansion [17, 18]. The general method for NK cell expansion is using unsorted PBMCs, CD3^+^ T-cell depletion, or NK cell isolation for allogeneic application with two sequential steps, CD3^+^ cell depletion and CD56^+^ cell enrichment, using immune-magnetic bead separation [19]. Subsequently, the ex vivo expansion of NK cells have used combinations of cytokines with or without feeder cells.

In this study, we obtained allogeneic primary NK (pNK) cells from PBMCs of healthy donors. Using expansion and cryopreservation, we aimed to analyze the potent anti-tumor activity of pNK cells both in vitro and in vivo*.*

## Materials and methods

### Isolation of human NK cells

Human PBMCs were collected from four healthy donors via leukapheresis. All healthy donors provided written informed consent prior to conducting the study. CD3^−^CD56^+^ pNK cells were obtained through CD3^−^/CD56^+^ selection with anti-CD3 (Miltenyi Biotec, Bergisch Gladbach, Germany) and anti-CD56 antibodies (Miltenyi Biotec) using the closed and automated platform, Prodigy (Miltenyi Biotec).

### Ex vivo expansion and cryopreservation of human pNK cells

The expansion of isolated pNK cell was performed with a GMP-compliant method at CHA Biotech (Seongnam, Korea). The cells were seeded on a γ-globulin (Greencross, Yongin, Korea) and anti-NKp46 (R&D Systems, Minneapolis, MN, USA)-coated flask and cultured in Alys505NK serum-free medium (CSTI, Sendai, Japan) supplemented with 1000 IU/mL recombinant human IL-2 (Novartis, Basel, Switzerland), 50 ng/mL recombinant human IL-18 (R&D system, Minneapolis, MN, USA), and 5% heat-inactivated autologous plasma. Fresh culture medium was added every 1 to 3 days depending on the cell density (2 × 10^6^ cells/mL). On Day 6, the cells were transferred to a culture bag (NIPRO, Osaka, Japan), and cultured for 14 days. The pNK cells were cryopreserved with Cryostor CS5 (BioLife Solutions, Bothell, WA, USA).

### Human cell lines

The human ovarian cancer cell lines (OVCAR3 and SKOV3), the human breast cancer cell lines (MCF7, MCF10A, MDA-MB-231, MDA-MB-468, BT-20, SK-BR3 and T-47D) and the leukemia cell line K562 were purchased from the American Type Culture Collection (ATCC, Manassas, VA, USA), and they were cultured under the conditions recommended by ATCC. A2780 and its cisplatin-resistant counterpart A2780cis were purchased from the European Collection of Authenticated Cell Cultures (ECACC), and they were maintained under the conditions recommended by ECACC. HLA-C genotypes for all cell lines are listed in Additional file: Table S1.

### Flow cytometry

The cells were stained using the antibodies in Additional file: Table S2 in the dark at 4 °C for 20 min. For intracellular staining, cells were stained with anti-Perforin-PE (eBiosciences, San Diego, CA, USA) or anti-Granzyme B-PE (eBiosciences), and they were fixed and permeabilized using BD CytoFix/CytoPerm™ (BD Biosciences). For evaluation of IFN-γ production, pNK cells were incubated in a 5% CO_2_ atmosphere with the addition of PMA/Ionomycin (BioLegend, San Diego, CA, USA) and BD GolgiPlug™ (BD Biosciences) at 37 °C for 4 h. Cells were stained with anti-CD3-FITC (eBiosciences) and anti-CD56-APC (eBiosciences), further stained with anti-IFN-γ-PE (eBiosciences), and fixed and permeabilized using BD CytoFix/CytoPerm™(BD Biosciences). Stained cells were analyzed using CytoFLEX flow cytometer (Beckman Coulter, Brea, CA, USA), and data was analyzed using FlowJo version 10.1 software (Treestar Inc., Ashland, OG, USA).

### CD107a degranulation assay

The pNK cells were cocultured with K562 at an Effector:Target (E:T) ratio of 5:1 and anti-CD107a-PE (eBiosciences) for 4 h. After coculturing, cells were stained with anti-CD3-FITC (eBiosciences) and anti-CD56-APC (eBiosciences). CD107a expressed on CD3^−^CD56^+^ pNK cells was analyzed using a CytoFLEX flow cytometer, and data were analyzed using FlowJo version 10.1 software.

### RNA sequencing

RNA of cryopreserved pNK cells at Day 0 and Day 14 was isolated using TRIzol reagent (Invitrogen, Carlsbad, CA, USA). To assess the integrity of total RNA, samples were run on the *TapeStation RNA screentape (Agilent, Santa Clara, CA, USA).* In order to construct cDNA libraries, we used the TruSeq Stranded mRNA LT Sample Prep Kit (Illumina Inc., San Diego, CA, USA). The protocol comprised polyA-selected RNA extraction, RNA fragmentation, random hexamer primed reverse transcription, and 100 nt paired-end sequencing using Illumina NovaSeq 6000 (Illumina, Inc.). The assembly of known transcripts was processed using StringTie v1.3.4d (Pertea, Mihaela, et al., 2015, 2016). The expression profiles were used to conduct additional analyses such as differentially expressed gene (DEG) analysis. Gene classification and gene ontology analyses were based on DAVID (http://david.abcc.ncifcrf.gov/) and Enrichr (https://maayanlab.cloud/Enrichr/) searches. RNA-seq data that support the findings of this study have been deposited in the Gene Expression Omnibus repository with the accession codes GSE163538 and GSE166933.

### Cytotoxicity assay

NK cell cytotoxicity against tumor cells was evaluated using carboxyfluorescein succinimidyl ester/7-aminoactinomycin D (CFSE/7-AAD) flow cytometry assay. After staining the target cells with CFSE (MA 02451, Thermo Fisher Scientific, Waltham, USA), pNK cells were mixed with CFSE-stained target cells at various E:T ratios incubated for 4 h. After re-suspending the cells in PBS with 7-AAD (Thermo Fisher Scientific), the percentage of target cell lysis was determined using CytoFLEX flow cytometer and FlowJo software. In blocking experiments, before the addition of target cells, NK cells were preincubated with the blocking antibodies listed in Additional file: Table S3 for 30 min.

### HLA-C mRNA expression in various cancer cell lines

For quantitative real-time PCR (RT-PCR) analysis, total RNA was prepared using TRIzol and cDNA was synthesized from 1 μg total RNA. Quantitative RT-PCR for *HLA-C1*, *HLA-C2*, and the housekeeping gene *GAPDH* was performed using iScript One-Step RT-PCR kit with SYBR Green (Bio-Rad, Hercules, CA, USA) according to manufacturer’s instructions. The primer sequences used in the experiment are listed in Additional file: Table S4.

### siRNA transfection for *HLA-C* knockdown

Validated HLA-C siRNAs and control siRNA (RNAi negative control duplex) were purchased from Bioneer (Daejeon, Republic of Korea). A2780 cells were transfected with siRNAs using Lipofectamine RNAiMAX (Invitrogen) according to manufacturer’s instructions.

### In vivo distribution study

Cryopreserved pNK cells were intravenously injected into BALB/c nude mice. Mice were sacrificed at 2, 24, 48, 72, and 168 h under general anesthesia. To isolate human cells, mouse organs, and blood genomic DNA, Tissue DNA Extraction Kit (D-1006, BioFactories,) and Blood DNA Extraction Kit (D-1001, BioFactories) were used according to manufacturer’s protocols. Mouse organ genomic DNA was isolated from the whole liver, lungs, spleen, ovary, kidney, bone marrow, heart, and blood. Quantitative PCR experiments were performed using an Applied Biosystems ViiATM 7 Real-time PCR instrument (Thermo Fisher Scientific). Human Alu amplification was performed using a 10 μL volume of commercial primers and probe (Hs00867356_s1, Thermo Fisher Scientific) Human genomic DNA was prepared by serial dilution, a standard curve was plotted, and human cell numbers were calculated.

### In vivo xenograft tumor model

Xenograft tumors were established in 7- to 8-week-old female NSG mice (JAbio, Suwon, Republic of Korea). A2780cis cells (4 × 10^6^) were subcutaneously inoculated in the right flank, tumor volume was calculated according to the following formula: volume = (width^2^ × length)/2. The animals were randomized into three treatment groups: (1) PBS, (2) cisplatin (1 mg/kg), (3) pNK (1 × 10^7^). Cisplatin was intraperitoneally injected to mice once a week for 4 weeks, and pNK cells were intravenously injected twice a week for 4 weeks. For distribution study in tumor, CFSE-stained pNK cells were intravenously (1 × 10^7^) injected into A2780cis tumor-bearing NSG mice after 5 weeks of tumor inoculation. Tumors were resected after 2, 24, and 72 h of pNK cell injection. Frozen tumor tissues were cut into 5 μM-thick sections, and they were fixed with ice-cold acetone for 10 min. Nuclei were stained with 4,6-diamidino-2-phenylindole (DAPI, Invitrogen) before mounting. Fluorescence signal was detected using a Zeiss LSM 510 microscope (Carl Zeiss Microscopy, Jena, Germany).

### ELISA

IFN-γ in cell culture supernatants was measured using Human IFN-γ ELISA Set (BD Biosciences, San Jose, CA, USA) according to the manufacturer’s protocol.

### Statistical analysis

Data are presented as the mean ± standard deviation (SD). Statistical analyses were performed using unpaired or paired Student’s *t*-tests with GraphPad Prism Software v.6 (GraphPad, La Jolla, CA, USA), and a *P*-value less than 0.05 was considered statistically significant. Data are represented as *P < 0.05, **P < 0.01, ***P < 0.001 and ****P < 0.0001.

## Results

### Characterization of ex vivo-expanded pNK cells for 14 days

The mean number of total mononuclear cells in the leukapheresis products from four donors was 2.11 ± 0.24 × 10^10^ cells, and the mean percentage of NK cells was 13.2 ± 4.15% (range, 7 ~ 18.7%). After isolation, an average of 1.05 ± 0.04 × 10^9^ cells (range, 1 ~ 1.1 × 10^9^) with an average of 94.9 ± 2.66% CD3^−^CD56^+^cells were obtained. The isolated pNK cells were divided into 6× 10^7^ cells/vial and cryopreserved.

**Fig. 1:**
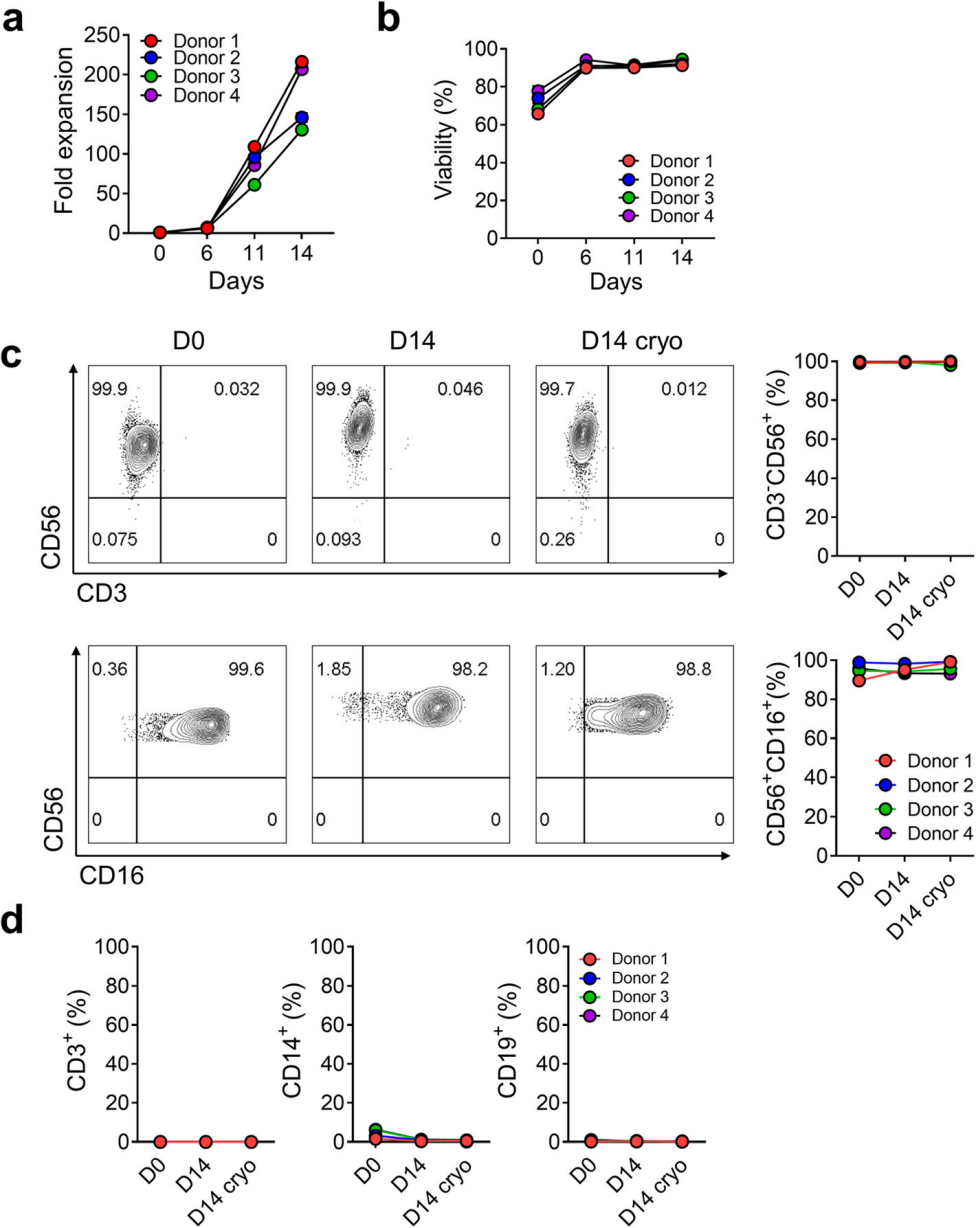
Characterization of *ex vivo*-expanded, cryopreserved pNK cells. **a.** The fold expansion of primary natural killer (pNK) cells from 4 different donors at 0, 6, 11, 14 days. **b.** The viability of expanded pNK cells was evaluated using propidium iodide staining. **c-d.** Identification of key immune NK cells following ex vivo expansion (D14) and cryopreservation (D14 cryo). Percentage of CD3^−^CD56^+^, CD56^+^CD16^+^, CD3^+^CD56^−^, and CD3^−^CD56^−^ cells (**c**). CD3^+^, CD14^+^, and CD19^+^ cells from four different donors were analyzed using flow cytometry (**d**)

Each vial of pNK cells from the four donors was expanded for 14 days with a mixture of antibodies and cytokines without feeder cells. The total cell numbers were measured on Days 0, 6, 11, and 14 during expansion. On Day 14, the expansion fold was 175 ± 43-fold (Fig. 1a) and the viability of expanded cells was 93 ± 1% (Fig. 1b). We analyzed the proportion of cells before culture(D0), after 14 days of culture(D14), and after cryopreservation (D14 cryo).

 The proportion of CD3^−^CD56^+^ pNK cells was 99.5 ± 0.4%, 99.7 ± 0.2%, and 99.9± 0.1% on D0, D14, and D14 cryo, respectively. CD56^+^CD16^+^ pNK cells were 94.7 ± 3.9%, 95.2 ± 2.1%, and 96.7 ± 3% on D0, D14, and D14 cryo, respectively (Fig. 1C). On D14, CD3^+^ T cells, CD14^+^ monocytes, and CD19^+^ B cells were 0%, 0.8 ± 0.3%, and 0.3 ± 0.1%, respectively. After freezing, no change was observed in cell distribution (Fig. 1d). On culturing pNK cells until 21 days, the total cell number continuously increased (4.9 ± 0.2 × 10^10^ cells at Day 21) and a high viability was maintained throughout (Additional file: Fig. S1a, b). The pNK cells at Day 21 also retained a high proportion (99.5%) of CD3^−^CD56^+^ cells (Additional file: Fig. S1c).

### Phenotypic comparisons of resting and expanded pNK cells

**Fig. 2:**
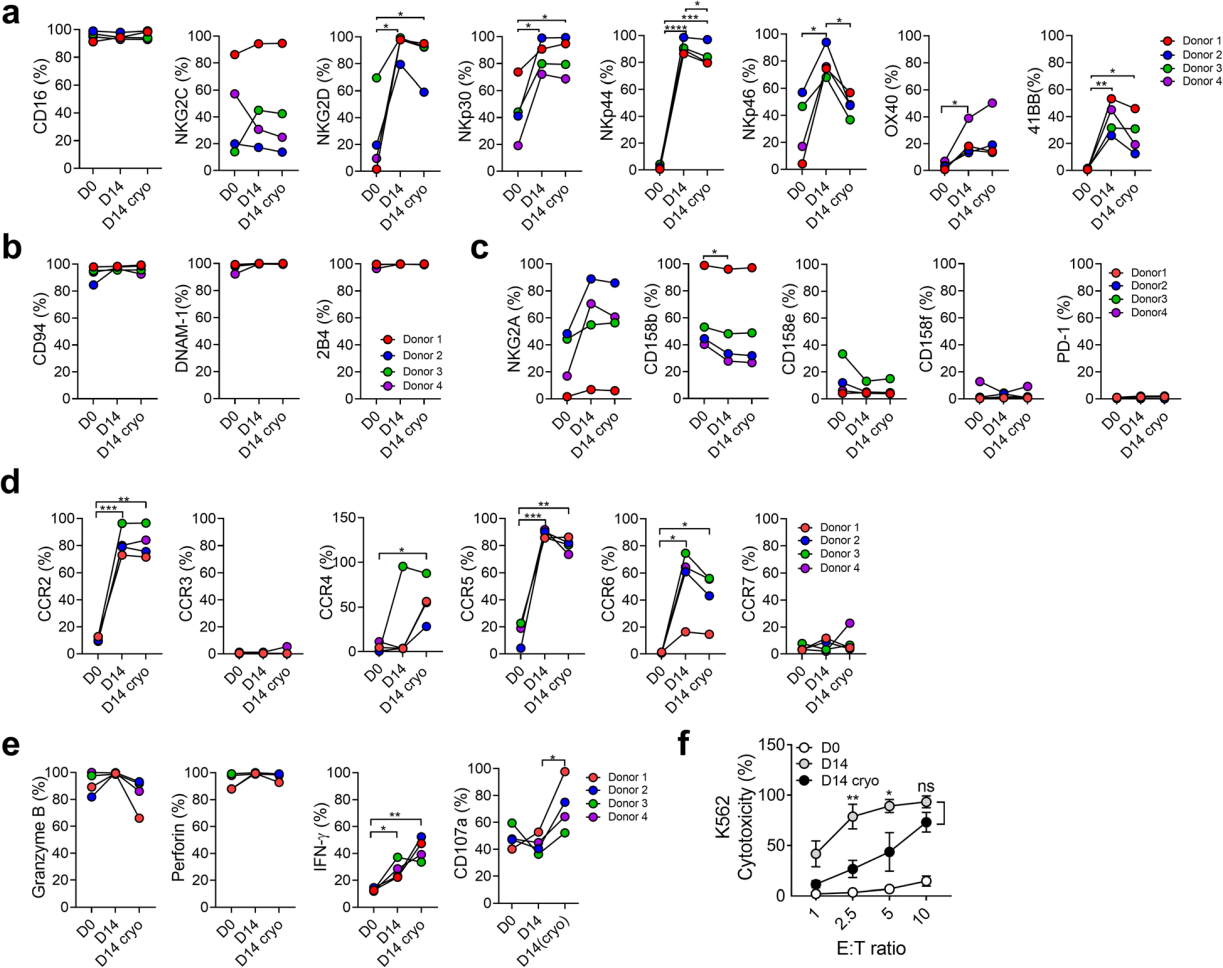
Phenotypic analysis of pNK cells before and after expansion and after cryopreservation. **a-e**. Surface expression of activating receptors (**a**), co-activating receptors (**b**), inhibitory receptors (**c**), chemokine receptors (**d**), cytolytic proteins, and functional markers (**e**) of pNK cells from 4 different donors was analyzed using flow cytometry before (D0) and after expansion (D14) and after cryopreservation (D14 cryo). CD107a expression were assessed after NK cells were co-cultured with K562 cells for 4 h at the E:T ratio of 1:1. Statistical analysis was performed using a paired *t*-test (*P < 0.05, **P < 0.01, ***P < 0.001, and ****P < 0.0001). D0 was compared with D14 or D14 cryo. Furthermore, D14 was compared with D14 cryo. **f.** pNK cells before (D0) and after (D14) NK cell expansion and after cryopreservation (D14 cryo) were cultured with K562 at the indicated E(Effector):T(Target) ratio for 4 h. pNK cell cytotoxicity against K562 was measured using the carboxyfluorescein succinimidyl ester/7-aminoactinomycin D (CFSE/7-AAD) assay. The assay was performed twice with expanded pNK cells from four different donors. Data are represented as mean ± SD. Statistical analysis was performed using two-way ANOVA (*P < 0.05 and **P < 0.01)

We evaluated various receptors of pNK cells on D0, D14, and D14 cryo using fluorescence-activated cell sorting (FACS) analysis. On D14, the expression of activating receptors such as NKG2D, NKp30, NKp44, and NKp46 was significantly increased, CD16 was maintained at a high level (95%), and NKG2C was increased in some donors as compared to those on D0. OX40 and 41BB slightly increased (Fig. 2a). Co-receptors such as CD94, DNAM-1, and 2B4 were significantly expressed from D0 to D14 (Fig. 2b). Before and after freezing, receptors such as CD16, NKG2C, NKp30, OX40, CD94, DNAM-1, and 2B4 showed an equivalent expression. NKG2D, NKp44, and 41BB showed slightly decreased expression levels; however, the changes in NKG2D and 41BB expression were not statistically significant. NKp46 expression, however, was significantly decreased. Inhibitory receptors such as CD158b and CD158e decreased on D14 than on D0, but only NKG2A slightly increased and CD158f did not change. Interestingly, the major immune check point protein, programmed cell death-1 (PD-1), did not increase in our culture medium (Fig. 2c). Additionally, some transcription factors involved in NK cell functions were analyzed. Eomes and GATA3 significantly increased during expansion while T-bet and E4BP4 were high since Day 0. The proliferation marker, Ki67, was significantly increased during expansion (Additional file: Fig. S2a). The expression of chemokine receptors such as CCR2, CCR5, and CCR6 significantly increased on D14 as compared to that on D0, CCR3 and CCR7 did not change, CCR4 increased, and CCR6 slightly decreased in some donors (Fig. 2d). CXCR3 and CXCR6 increased whereas CXCR1 and CXCR4 decreased after expansion (Additional file: Fig. S2b). Expanded pNK cells significantly expressed adhesion molecules, such as CD2, CD11a, CD18, ITGA1, and ITGB7, except CD62L (Additional file: Fig. S2c). CD69, an activation marker, was expressed shortly after NK cell activation, and it increased after expansion while CD57, a senescence marker [20, 21], decreased after expansion (Additional file: Fig. S2d). Cytolytic proteins such as perforin and granzyme B were significantly expressed from D0 to D14, and the levels were maintained after freezing. The expression of IFN-γ was slightly increased. CD107a degranulation did not change significantly, but it was increased after cryopreservation (Fig. 2e).

Finally, we evaluated the cytotoxicity of pNK cells against K562 cells at D0, D14, and D14 cryo. Cytotoxicity against K562 was significantly increased on D14 than on D0 (14.5 ± 3.5% vs. 93.2 ± 4.1% at an E:T ratio 10:1, *P* < 0.05), and it was maintained after freezing at an E:T ratio of 10:1 (Fig. 2f). The cytotoxicity of 21 days-expanded pNK cells against K562 was similar to that of 14 days-cultured pNK cells at E:T ratios of 5:1, 10:1, and 20:1 (Additional file: Fig. S1d).

### Gene expression signature of ex vivo-expanded pNK cells using RNA sequencing

**Fig. 3:**
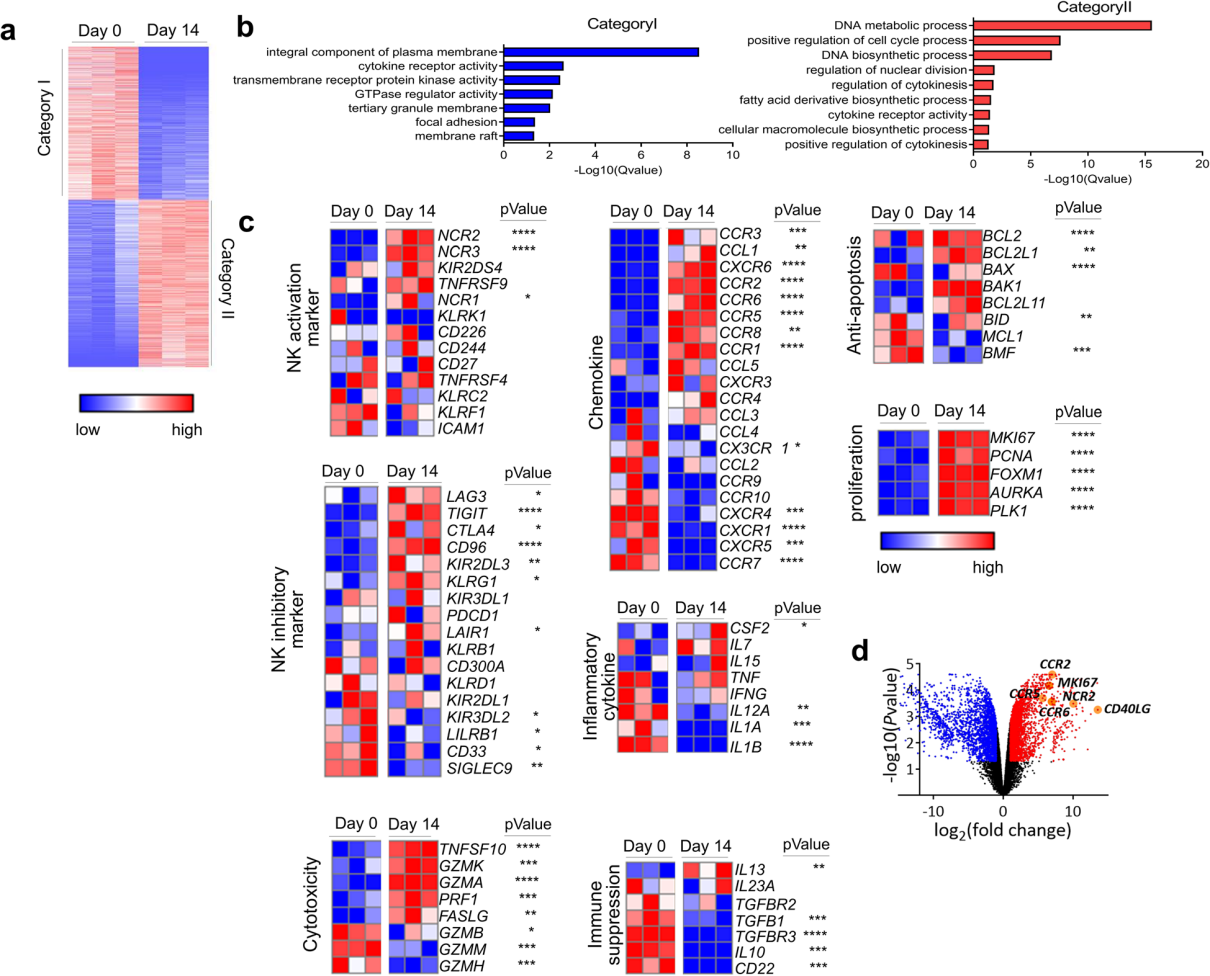
Gene expression signature of cryopreserved pNK cells before or after expansion using RNA sequencing. Genome-wide transcriptional profiles of cryopreserved pNK cells before (Day 0) or after expansion (Day 14). **a.** The heatmap depicts clustering of 5471 differentially expressed genes on Day 14 as compared to Day 0 pNK cells (q-value< 0.05 and fold change> 2 or < 0.5). Each column represents the expression profiles of individual donors in each experimental group. Upregulated gene set at Day 0 (Category I) and Day 14 (Category II). Warm color (red) denotes an increase in gene expression whereas cold color (blue) indicates a decrease as compared to the average level of gene expression in Day 0 pNK cells. **b.** Gene Ontology Biological Process (GOBP) analysis of upregulated genes on Day 14 (q-value< 0.05). **c.** Heat maps representing genes (labels to the right of plots) clustered in GOBP NK activation, NK inhibition, cytotoxicity, chemokine, inflammatory cytokine, immune suppression, anti-apoptosis, and proliferation (labels to the left of plots) with scaled intensities. Warm color (red) denotes an increase in gene expression whereas cold color (blue) indicates a decrease as compared to the average level of gene expression in Day 0 pNK cells. **d.** Volcano plot of the RNA-seq data (q-value< 0.05 and fold change> 2 or < 0.5)

We performed RNA sequencing to employ an unbiased approach to identify the altered gene expression enhancing the functions of ex vivo-expanded pNK cells for 14 days, and we transcriptionally compared Day 0 and Day 14 pNK cells. In total, 5471 genes were differentially expressed between Day 0 and Day 14 pNK cells with 2855 upregulated (Category I) and 2616 downregulated genes (Category II) on Day 14 as compared to those on Day 0 (Fig. 3a). Gene Ontology Biological Process (GOBP) analysis results revealed that the upregulated DEGs were enriched in DNA metabolic processes, cell cycle regulation, DNA biosynthesis, nuclear division regulation, and cytokine regulation (Fig. 3b).

The NK activation markers, *NCR2* (1064-fold), *NCR3* (5-fold), *KIR2DS4* (2-fold), and *TNFRSF9* (2-fold), were increased on Day 14 pNK cells than on Day 0 pNK cells (Fig. 3c). Concomitantly, the genes related to cytotoxicity (*TNFSF10, GZMK*, *GZMA*, *PRF1*, and *FASLG*), chemokines (*CCR1*, *CCR2*, *CCR5,* and *CCR6*), anti-apoptosis (*BAX*, *BCL2,* and *BCL2L1*), and cell proliferation (*MKI67*, *PCNA*, *FOXM1*, *AURKA,* and *PLK1*) were enriched on Day 14 (Fig. 3c). Several genes involved in immune suppression, including *CD22*, *IL-10*, *TGFβ*, and *TGFβR3*, were also decreased on Day 14. PD-1, which was only slightly expressed (1%) on Day 0 and Day 14 pNK cells using FACS analysis (Fig. 2c), was not detected in the RNA sequencing data. Among the significantly expressed transcripts, *CD40L* and *CCR5* were increased by 12,018-fold and 49-fold on Day 14 pNK cells than on Day 0 pNK cells (Fig. 3d).

To investigate whether CD3^−^CD56^+^ selection leads to gene expression alterations, we performed RNA sequencing on Day 0 and Day 14 samples of PBMCs without CD3^−^CD56^+^ selection (non-selectively expanded pNK cells) after an ex vivo expansion of cells using the same manufacturing method (Additional file: Fig. S3a-3c). Similar to the results of selectively-expanded pNK cells (Fig. 3), NK activation markers, cytotoxicity-related genes, chemokines, anti-apoptosis, and cell proliferation were enriched in non-selectively expanded pNK cells on Day 14 than on Day 0 (Additional file: Fig. S3). However, the increase of some activating receptors, chemokines, and anti-apoptotic genes, such as *NKp44* (1064-fold), *TNFSF10* (24.5-fold), *CCR2* (130.1-fold), *CCR3* (1427.5-fold), *CCR5* (49.9-fold)*, CCR6* (124.1-fold), *and BCL2* (10.5-fold), was more significant in selectively expanded pNK cells than in non-selectively expanded pNK cells (6.3-, 9.7-, 6.6-, NA, 16.7-, NA, and 1.7-fold, respectively) (Fig. 3 and Additional file: Fig. S3).

These results indicated that the NK cell culture method from PBMCs with or without CD3^−^CD56^+^ selection increased the expression of various activating NK receptors and cytotoxicity-related genes and when pNK cells were cultured with CD3^−^CD56^+^ selection, CD40L, NKp44, and migration-related genes were more significantly expressed.

### Cytotoxicity of expanded pNK cells against various ovarian and breast cancer cell lines

**Fig. 4:**
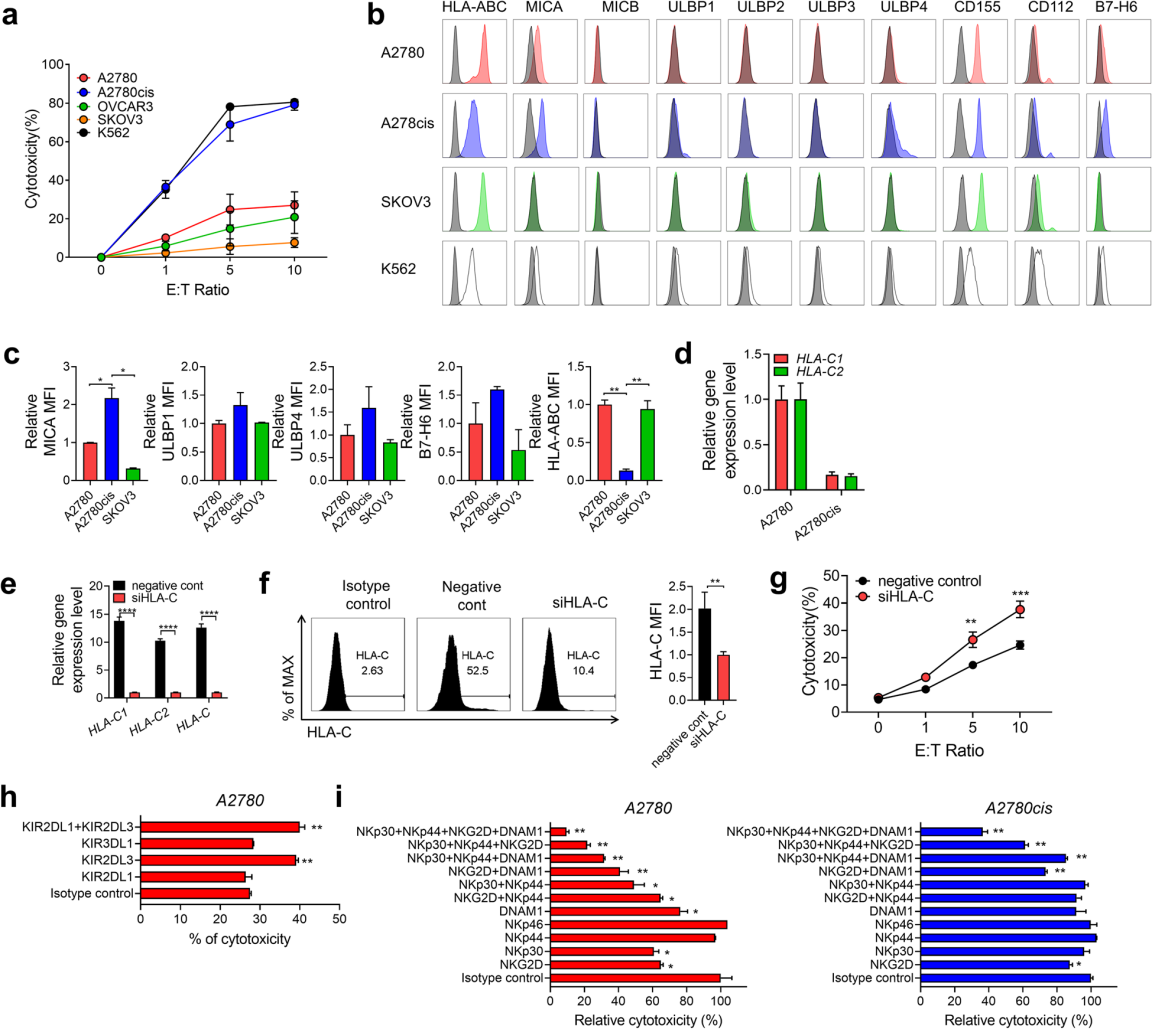
Cytotoxicity of cryopreserved pNK cells against ovarian cancer cell lines. **a.** Cytotoxicity of cryopreserved pNK cells against various ovarian cancer cell lines was analyzed using CFSE/7-AAD assay with the indicated E:T ratio. **b-c**. Expression of HLA-class I, ULBP-1, ULBP-2, MIC-A/B, CD112, and CD155 in three different ovarian cancer cell lines was analyzed using flow cytometry. Representative histograms of the expression of receptors in each cell lines (**b**). Grey histograms represent isotype controls. Expression intensity of MICA, ULBP1, ULBP4, B7-H6, and HLA-ABC in three different ovarian cancer cell lines. Mean fluorescence intensity determined using flow cytometry (**c**). Data represented as mean ± SD. Statistical analysis was performed using an unpaired *t*-test (*P < 0.05 and **P < 0.01). **d.** Expression of HLA-C1 and HLA-C2 mRNA in A2780 and A2780cis cells. Data are represented as mean ± SD. **e-f.** Efficiency of siRNA treatment of HLA-C on A2780 cells. Gene knockdown efficiencies were obtained using quantitative RT-PCR (**e**) and FACS (f). Data are represented as mean ± SD. Statistical analysis was performed using an unpaired *t*-test (**P < 0.01 and ****P < 0.0001). **g.** Cytotoxicity of cryopreserved pNK cells against A2780 cells with and without HLA-C knockdown was analyzed using CFSE/7-AAD assay with the indicated E:T ratio. **h.** Cryopreserved pNK cells were pre-incubated with blocking antibodies against KIR2DL1, KIR2DL3, and KIR3DL1, and cytotoxicity was analyzed against A2780 using CFSE/7-AAD assay in triplicate. E:T ratio was 10:1. Each group was compared to the isotype control. Statistical analysis was performed using an unpaired *t*-test (**P < 0.01). **i.** Cryopreserved pNK cells were pre-incubated with blocking antibodies against NKG2D, NKp30, NKp44, and DNAM-1, and cytotoxicity was analyzed against A2780 or A2780cis cells using CFSE/7-AAD assay in triplicate. E:T ratio was 10:1. Percentage inhibition of cytotoxicity was calculated as the percentage of inhibition via the isotype control antibody. Data are represented as mean ± SD. Each group was compared to the isotype control. Statistical analysis was performed using an unpaired *t*-test (*P < 0.05 and **P < 0.01)

Next, the cytotoxicity of expanded pNK cells was evaluated in vitro against human ovarian cancer cell lines. Various tumor cell lines displayed different levels of susceptibility to cytolysis to expanded pNK cells (Fig. 4a). To understand this varied susceptibility, expression of different ligands for NK receptors was analyzed on cancer cell lines. In line with the cytotoxicity data, the pNK-sensitive A2780cis cells showed high expression levels of NKG2D ligands, such as MICA, ULBP1, ULBP4, and NKp30 ligand (B7-H6), as compared to the pNK-non-sensitive SKOV3 cells.

In contrast, the expression levels of inhibitory KIR ligand (HLA-ABC) were significantly lower in A2780cis cells than in pNK-non-sensitive cancer cells (A2780 and SKOV3 cells) (Fig. 4b, c). As CD158b (KIR2DL2/3) was increased in expanded pNK cells (Fig. 2c), we investigated the effect of HLA-C1 or C2 expression on the cytotoxicity of pNK cells. Gene expression levels of both *HLA-C1* and *HLA-C2* were significantly decreased in pNK-sensitive A2780cis cancer cells (approximately 0.17-fold of the level in A2780 cells) (Fig. 4d) although both A2780 and its cisplatin-resistant A2780cis subline possessed HLA-C1/C2 genotypes (Additional file: Table S1). To elucidate the contribution of HLA-C1 and -C2 expression in NK alloreactivity, we introduced siRNAs for *HLA-C* in A2780 cells. After confirming that mRNA and protein expression of both HLA-C1 and HLA-C2 significantly decreased after HLA-C siRNA transfection (Fig. 4e, f), we verified that the cytotoxicity of pNK cells was significantly increased in A2780 cells after HLA-C siRNA transfection (20% vs. 40% at an E:T ratio of 10:1, *P* < 0.001, Fig. 4g). Additionally, we also found that pNK cytotoxicity was increased after KIR2DL3 blockade rather than other inhibitory KIR blockade (Fig. 4h). Collectively, these results suggested that inhibitory KIR-KIR ligand interaction, especially KIR2DL3-HLAC1, effectively contributed to pNK cell alloreactivity. When we examined HLA-G expression in these ovarian cancer cell lines to assess its possible inhibitory function on our pNK cells, we did not find the increase of HLA-G expression (Additional file: Fig. S4).

To evaluate the role of activating NK receptors, a cytotoxic assay was performed with expanded pNK cells in the presence of blocking antibodies specific to NKG2D, NKp30, NKp44, NKp46, and DNAM-1. Although blocking a single receptor alone slightly affected the cytotoxicity, NKG2D blockade mostly inhibited NK killing as compared to any other single blocking. In contrast, blocking multiple receptors led to a substantial reduction in cytotoxicity. Particularly, blocking all four receptors led to approximately 90% blocking in A2780 and 60% blocking in A2780cis cells than in IgG control (Fig. 4i).

We also evaluated the cytotoxicity and IFN-γ secretion of expanded pNK cells against human breast cancer cell lines (Additional file: Fig. S5a & S5b), revealing that MCF-7 showed the highest sensitivity to our pNK. Unlike ovarian cancer cell lines, expression levels of NKG2D ligands were similar in both pNK-sensitive target cells (MCF-7) and pNK-non-sensitive target cells (MDA-MB-231) (Additional file: Fig. S5c). However, pNK-non-sensitive cancer cells (MDA-MB-231and MDA-MB-468) expressed high levels of *HLA-C2* (Additional file: Fig. S5d) whereas pNK-sensitive cancer cells (MCF-7) expressed low levels of both *HLA-C1* and *HLA-C2*, indicating the contribution of inhibitory KIR-KIR ligand mismatch in pNK cytotoxicity. In the blocking analysis of MCF7 cancer cells, blocking a single receptor alone except for NKp46 affected the cytotoxicity as much as blocking multiple receptors (Additional file: Fig. S5e).

Taken together, these results demonstrated that expanded pNK cells have the ability to kill a wide range of tumor cells, and the process is not only regulated by activating receptor-ligand interaction but also inhibitory receptor-ligand interaction.

### In vivo distribution of ex vivo-expanded pNK cells

**Fig. 5:**
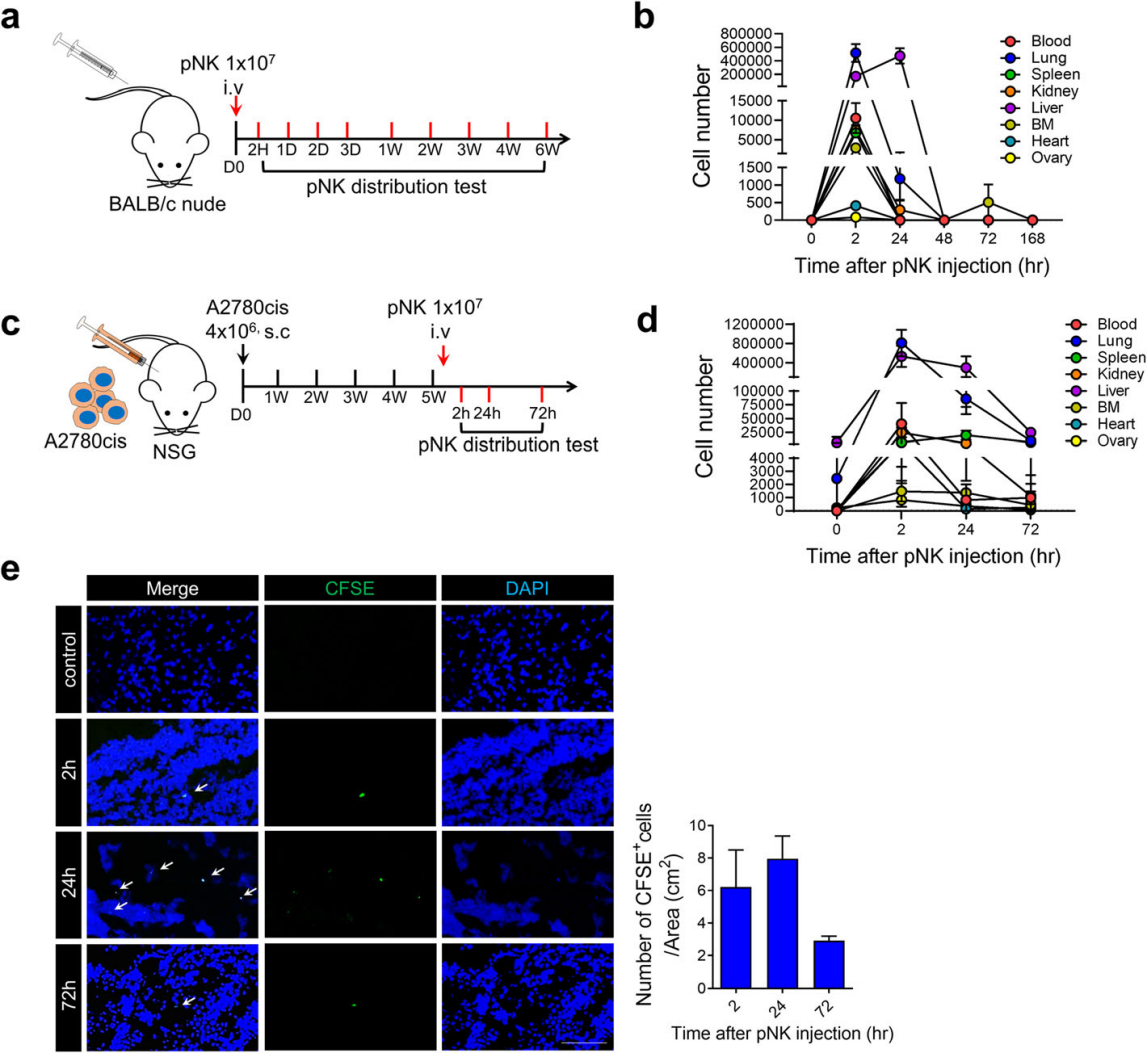
In vivo distribution of cryopreserved pNK cells. **a-b.** Cryopreserved pNK cells (1 × 10^7^ cells) were intravenously injected into Balb/c nude mice. Mice were sacrificed at 2, 24, 48, 72, and 168 h (*n* = 3 per time point) (**a**). The numbers of intravenously injected NK cells calculated from the amount of human genomic DNA extracted from the blood, lungs, spleen, kidney, liver, bone marrow, heart, and ovary were quantified using Alu qPCR (**b**). Data are represented as mean ± SD. **c-e**. Expanded pNK cells (1 × 10^7^ cells) were intravenously injected into A2780 tumor-bearing NSG mice after 40 days of tumor inoculation. Tumor were resected 2, 24, and 72 h after injection of pNK cells (n = 3 per time point) (**c**). The number of intravenously injected pNK cells was calculated from the amount of human genomic DNA extracted from the tumors using Alu qPCR (**d**). Immunofluorescence staining for transferred cells (CFSE, green) and nuclei (DAPI, blue) in tumor tissues (**e**). Scale bars, 100 μm. Left panel: Representative images of intravenously pNK injected mice tumor. Right panel: The number of pNK cells in tumors was measured (n = 3 per group). Data are represented as mean ± SD

To investigate the in vivo distribution of cryopreserved pNK cells, we intravenously administered pNK cells (1 × 10^7^ cells/mouse) to BALB/c nude mice, and the mice were sacrificed at each post-injection time point until 168 h (Fig. 5a). Intravenously-injected pNK cells first appeared in the blood, lungs, spleen, kidney, liver, heart, and ovary at 2 h, where they resided for 24 h and then gradually disappeared until 48 h (Fig. 5b).

To evaluate pNK cell distribution in tumor-bearing immune incompetent mice, CFSE-stained pNK cells (1 × 10^7^ cells/mouse) were intravenously injected into A2780cis tumor-bearing NSG mice after 40 days of tumor inoculation, and mice were sacrificed at 2, 24, and 72 h after pNK cell injection (Fig. 5c). Intravenously-injected pNK cells first appeared in the blood, lungs, spleen, kidney, liver, heart, ovary, and tumor at 2 h, and they were detected in the blood, lungs, spleen, and tumor until 72 h after injection (Fig. 5d, e). At 72 h, we observed 997 ± 343, 9657 ± 3634, and 7108 ± 3580 pNK cells in the blood, lungs, and spleen using qPCR. We also found several pNK cells (6.22 ± 2.28, 7.96 ± 1.39, and 2.92 ± 0.27 cells/cm^2^ at 2, 24, and 72 h, respectively) in the tumor sections via counting of CFSE-stained pNK cells.

### In vivo therapeutic effects of ex vivo-expanded day 14 pNK cells against human ovarian cancer cell line xenograft tumors

**Fig. 6:**
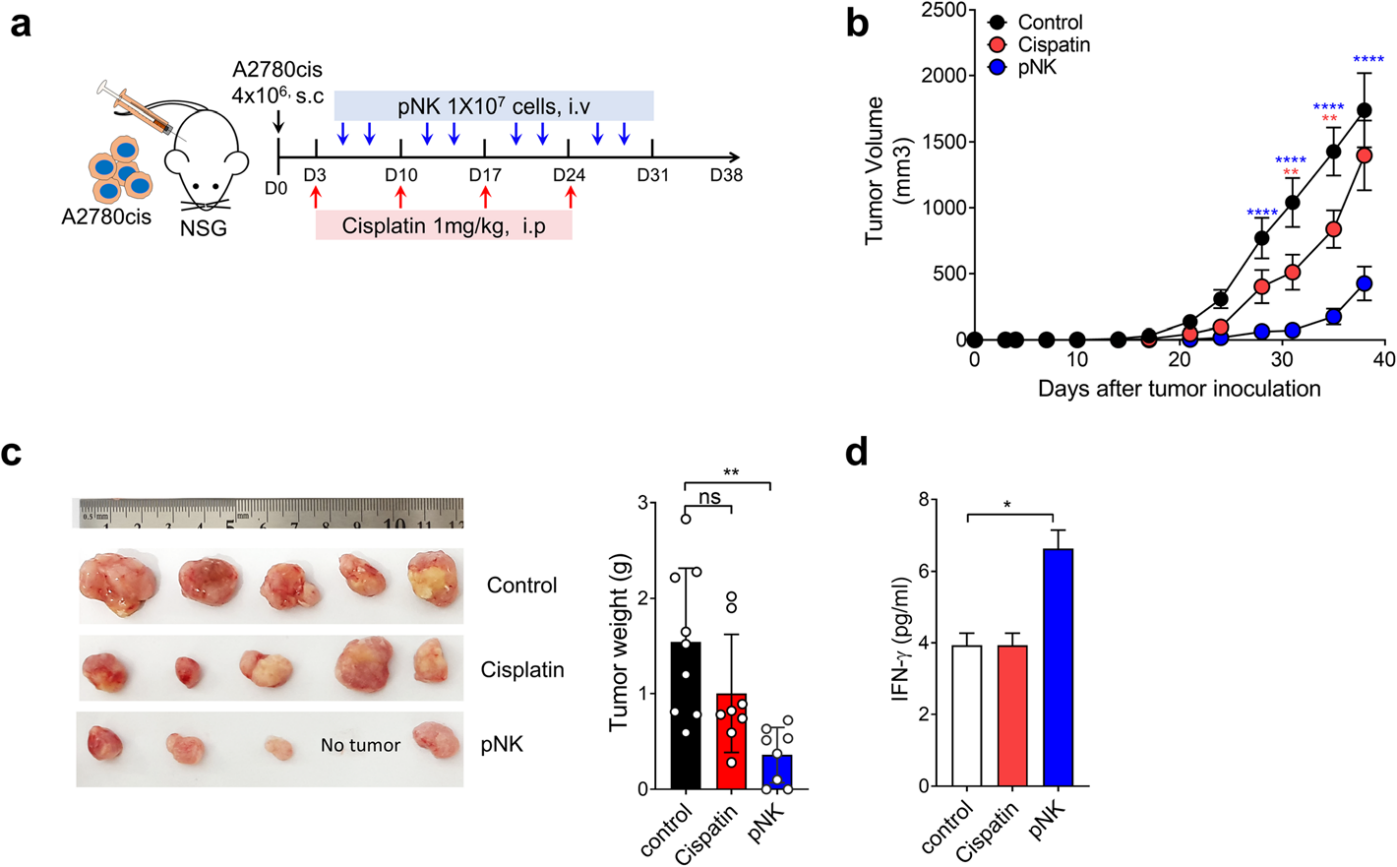
In vivo therapeutic effects of cryopreserved pNK cells against cisplatin-resistant A2780cis xenograft tumors. **a.** Experimental design for cisplatin and pNK cell treatment of xenograft mouse models bearing A2780cis cells. Cisplatin (1 mg/kg) was intraperitoneally injected into mice once a week (red arrow), and 1 × 10^7^ pNK cells were intravenously injected into mice on the indicated day (blue arrow) (*n* = 8–9 per each group). **b.** Tumor volume of A2780cis was measured at the indicated days after tumor injection. Statistical analysis was performed using two-way ANOVA (**P < 0.01 and ****P < 0.0001 as compared to the control group). The results are expressed as average ±SEM. **c.** Tumor weight was measured after the mouse was sacrificed. Left panel: Representative image of A2780cis tumor from each group mice sacrificed after 40 days of tumor injection. Right panel: Average tumor weight with SD (n = 8 per group). Each group was compared to the control. Statistical analysis was performed using an unpaired *t*-test (**P < 0.01). **d.** Interferon-γ levels in plasma samples from each group of mice. Plasma was harvested at Day 40 post-tumor injection. Each group was compared to the control. Statistical analysis was performed using an unpaired *t*-test (*P < 0.05)

To confirm the therapeutic efficacy of cryopreserved pNK cells in vivo, we established A2780cis xenograft on NSG mice. On Day 3, following subcutaneous tumor inoculation, mice were intravenously injected with 1 × 10^7^ cells/mouse pNK cells twice a week or intraperitoneally injected with cisplatin (1 mg/kg) once a week (Fig. 6a). In line with in vitro results, on Day 40, pNK cells induced a significant inhibition of A2780cis tumor progression (76%) as compared to the control whereas cisplatin treatment did not show a significant effect (16% reduction) (Fig. 6b, c). When we sacrificed the mice, the serum levels of IFN-γ were elevated, and they were 1.6-fold higher in mice treated with expanded pNK cells than in the control group (Fig. 6d). To evaluate the effect of HLA-C expression in cancer cells on the anti-tumor effects of pNK cells in vivo, we performed in vivo efficacy tests and compared the results with those of subcutaneous (s.c.) xenograft models in NSG mice using an HLA-C-high ovarian cell line (A2780) and its cisplatin-resistant subline (A2780cis), in which HLA-C expression was notably lower than that in its parental cell line (A2780) (Additional file: Fig. S6). To compare the models under similar conditions as far as possible, we injected pNK cells (1 × 10^7^ cells, weekly) after the tumors reached a size of 90–100 mm^3^ in both models, and tumor size was monitored until the mice were sacrificed after 15 days. The tumor growth in HLA-C-high A2780 xenografts was inhibited compared to that in the control (50% reduction compared to that in the control); however, the inhibition was notably lesser than that observed in HLA-C-low A2780cis (80% reduction compared to that in the control; P * < 0.05). These results were consistent with the in vitro results, in which the cytotoxicity of our pNK cells to A2780cis was markedly higher than that to A2780 (79.13 ± 2.79% vs 27.01 ± 6.95% at E:T ratio = 10:1). Taken together, these data suggest that the growth of ovarian cancer cells resistant to cisplatin was significantly inhibited by pNK treatment and this effect could be related to the low HLA-C expression in the cisplatin-resistant cells.

## Discussion

In this study, we produced GMP grade-ex vivo-expanded allogeneic pNK cells from leukapheresis products of healthy donors, and we characterized these pNK cells after cryopreservation. Furthermore, we created genome-wide transcriptome profiles of cryopreserved pNK cells using RNA-Seq. Using in vitro cytotoxicity and in vivo xenograft experiments, we also demonstrated the possible use of our pNK cells for treating chemoresistant ovarian cancers.

Cancer immunotherapy has become an increasingly important treatment option for various cancers. Although the anti-tumor activity of NK cells has been previously explored in several animal experiments, its transfer to humans is much more recent. Different ex vivo-expanded NK cells in various culture conditions exhibit some common characteristics. However, NK cells exhibit various activating states and different expression patterns depending on the culture conditions.

The culture conditions employed in our study led to expand the highly purified NK cells, although the expansion rate of NK cells obtained via our method was lower than that of NK cells cultured with autologous PBMC feeder cells. Our NK cell expansion rate was similar to that of CD3-depleted PBMCs in feeder-free culture conditions, even though the expansion rate of purified CD3^−^CD56^+^ NK cells is generally considered to be lower than that of CD3-depleted PBMCs (Additional file: Table S5). When we cultured different cells from PBMCs via our culture method, we obtained 1545-fold and 1000-fold expansion of NK cells for PBMCs and CD3-depleted PBMCs, respectively; these rates are comparable to those obtained in previous studies of NK cell expansion with feeder cells (Additional file: Table S6).

With regard to the representative gene expression patterns, our pNK cells characteristically showed high levels of NKp44 (91.28 ± 5.2%) and CCR5 (88.15 ± 3.21%) expression. However, NKp44 expression levels in NK cells expanded using other methods were variable and CCR5 expression was notably lower (< 5%) than that observed via our method, although only one study with CD3^+^ T cell-depleted PBMCs examined the expression of CCR5.

Cancer patients usually undergo a variety of standard treatments before receiving immunotherapy, and it is important to deeply understand the characteristics of NK cells in order to select the correct clinical setting for NK cell therapy.

Cryopreservation of NK cells is essential for the development of a readily-accessible off-the-shelf product for clinical use [22]. In contrast to other lymphocytes, NK cells are highly sensitive to freezing and thawing, resulting in the loss of NK cell activity on thawing [23, 24]. In this study, we showed that the cytotoxicity and phenotypes of all NK activating receptors, except NKp46, were maintained after thawing. Although the percentage of NKp46^+^ NK cells was significantly decreased following cryopreservation, NKp46 does not seem to be an important receptor for pNK cell cytotoxicity because blocking NKp46 did not influence the cytotoxicity of pNK cells in our study.

Although some previous studies have investigated the single-cell RNA sequencing of NK cells due to their heterogeneity, little is known about the transcriptome of NK cells after ex vivo expansion and cryopreservation. In this study, we observed that *CD40L* expression was altered the most in ex vivo-expanded and cryopreserved pNK cells as compared to that before expansion. CD40 ligand (CD40L), a member of the TNF superfamily of molecules, is a protein that is primarily expressed on activated T cells. CD40L has been reported to be upregulated on the surface of human and mouse NK cells on activation with IL-2 [25]. It has been demonstrated that IL-2/CD40 agonist combination therapy results in NK cell infiltration into mesothelioma tumors. NK cells do not play an effector role in this system but rather help in establishing a long-term memory response [26]. After examining the interplay between human dendritic and NK cells, another study reported that CD40/CD40L interaction increases the cytotoxic functions of NK cells [27] and leads to an inhibition of tumor cell growth in many human cancers.

The expression of CCR chemokine receptor genes was highly increased using our culture method, and the expression of CCR1, CCR2, CCR3, CCR5, and CCR6 was higher in pNK cells (approximately 3.97-, 130-, 1427-, 49-, 124-fold, respectively) than in the cells produced using any other culture method (approximately 0.7-, 4.1-, 0.13-, 18.58-, 3.4-fold, respectively) [28]. A recent study reported that CCR5-overexpressing NK cells enhance the antitumor effects in vivo [29]. These reports suggest that the CCR5-CCL5 interaction is important for NK cell homing to tumor sites and antitumor activity of NK cells. In contrast to the low expression (< 5% of NK cells) of CCR5 in other studies [30], significantly increased RNA and protein levels of *CCR5* were observed in our pNK cells in this study. Taken together, the results suggest that our pNK cells with a high expression of CD40L and CCR5 may induce high cytotoxic effects on CD40-high cancers, such as melanoma, breast, and head and neck cancers [31, 32], or CCL5-high cancers, such as ovarian, metastatic breast, cervical, and gastric cancers [33, 34].

Additionally, the pNK cells showed a significant increase in NKp44-activating receptor expression, which might induce a high efficacy against cancers with a high expression of NKp44 ligand, MML5, or PDGF, such as glioma, gastro-intestinal stromal tumor, and triple-negative breast cancer [35]. Although further in vivo studies are needed to verify the effect of CD40L, CCR5, and NKp44 expression on pNK cells on NK cell cytotoxicity against corresponding ligand-expressing tumor cells, finding candidates based on transcript profiling provides valuable information to develop new therapeutic options for pNK cell therapy.

Previously, many studies have reported the benefits of selecting an inhibitory KIR-KIRL-mismatched donor in the treatment of adult acute myeloid leukemia [12, 36]. Consistent with previous studies, we found the relevance of KIR-KIRL mismatch in NK cell cytotoxicity against several cancer cell lines. In the present study, we demonstrated that HLA-C knockdown by siRNA treatment induced a significant increase in pNK cytotoxicity against A2780 ovarian cancer cells, with a high expression level of *HLA-C1* and *HLA-C2* and low pNK cytotoxicity, implying that the alloreactivity of pNK cells is related to inhibitory KIR-KIRL mismatch. Considering the report that KIR2DL2/3^+^ NK cells rather than KIR2DL1^+^ or KIR3DL1^+^ NK cells are predominantly detected in the blood of patients after transplantation [37], and our result that pNK cells with high expression of KIR2DL2/3^+^ increased the cytotoxic activity after KIR2DL3 blocking, pNK cell therapy would lead to higher effectiveness by the selection of optimal patients with low *HLA-C* expression in tumors.

Expression of PD-L1 in tumor cells increases after platinum-based neoadjuvant chemotherapy in patients with non-small cell lung cancer (NSCLC) [38]. It has also been reported that cisplatin-resistant cancer cells express high levels of PD-L1 as compared to parent cells [39]. Some cisplatin resistance in NSCLC can also be accompanied by a resistance to NK-mediated immune responses in both primary NK cells and NK cell lines [39, 40]. Consistent with previous reports, the high expression of PD-L1 in A2780cis cells might be related to the resistance of A2780cis cells to NK92MI cell line. NK92MI cells, showing a higher PD-1 expression, demonstrated lower cytotoxicity to A2780cis cells compared with our pNK cells which showed low PD-1 expression (Additional file: Fig. S7). It can be assumed that the low PD-1 expression of our pNK cells contributes to the increased cytotoxicity of pNK cells against cisplatin-resistant cancer cell lines. Additionally, the expression levels of NK cell-activating receptors (NKp44, NKp46, DNAM-1 and CD16) of NK92MI are lower than those of our pNK cells according to a previous study [41]. Therefore, the pNK cells in this study can provide many advantages for the treatment of PD-L1-increased cisplatin-resistant tumors.

In addition, our expanded pNK cells demonstrated high proportion (99.5%) of CD3^−^CD56^+^ cells without T-cells. Considering that the critical side effect of allogenic NK immunotherapy in human is Graft-versus-host disease caused by alloreactive T-cells, or a passenger lymphocyte syndrome caused by donor-derived B –cells, the high purification of NK cells is very important to avoid side effect in clinical use [42, 43]. In this regard, our allogenic pNK cells have an advantage for clinical use.

We demonstrated here that our cryopreserved pNK cells induced a significant tumor growth inhibition (76%) in an in vivo cisplatin-resistant A2780cis xenograft model and high in vitro cytotoxicity whereas cisplatin induced a significantly less tumor inhibition (16%). We also confirmed that the high pNK cytotoxicity was related to an inhibitory KIR-KIRL mismatch via a downregulation of HLA-C1 and -C2 expression and upregulation of NKG2D ligand expression, such as MICA and ULBP4 expression. The pNK cell cytotoxicity against breast cancer cell lines was related to HLA-C expression rather than NKG2D ligand expression. Thus, it seems that KIR-KIRL mismatch highly affects pNK cytotoxicity than activating NKR ligand expression.

## Conclusion

In the present study, for the first time, we revealed a comprehensive genetic, phenotypic, and functional signature of ex vivo-expanded, cryopreserved pNK cells, which provides researchers the important information necessary to make appropriate choices for the treatment of human diseases using NK cells. Additionally, this study provides a rationale for the production of ex vivo-expanded, cryopreserved pNK cells using a feeder-free, GMP-compliant method. The generated pNK cells can be an effective immunotherapeutic strategy for chemoresistant ovarian cancers related to KIR-KIRL mismatch.

## Supplementary Information

**Additional file 1.**

## Data Availability

All data generated or analyzed during this study are included either in this article or in the supplementary Materials and Methods, Tables, Fig.s, and Fig. Legends files.

## Abbreviations

CFSE: Carboxyfluorescein succinimidyl ester
E:T: Effector: target
IFN-γ: Interferon-gamma
IL: Interleukin
KIR: Killer cell immunoglobulin-like receptor
MHC: Major histocompatibility complex
NCRs: Natural cytotoxicity receptors
NK cells: Natural killer cells
NKG2D: Natural killer group 2D
PBMCs: Peripheral blood mononuclear cell
PBS: Phosphate buffered saline
PD-1: Programmed cell death protein 1
PD-L1: Programmed cell death ligand 1
