# Supplementary material for: Ex vivo expanded allogeneic natural killer cells have potent cytolytic activity against cancer cells through different receptor-ligand interactions
Source: J Exp Clin Cancer Res. 2021 Oct 23;40:333. doi: 10.1186/s13046-021-02089-0 (PMC8539797; doi:10.1186/s13046-021-02089-0)
Supplement: Supplementary file 1 — Additional file 1. [file 13046_2021_2089_MOESM1_ESM.docx]

***Supplementary Materials and methods***

***Intracellular staining***

NK cells were stained with anti-CD3-eFluor 450 (eBioscience, San Diego, CA, USA) and anti-CD56-APC (eBioscience) or anti-CD56-PE (eBioscience) without any treatment. For intracellular staining, Cells were stained using the antibodies listed in Additional file: Table S6 and fixed and permeabilized using BD CytoFix/CytoPerm™ (BD Biosciences, San Jose, CA, USA). Stained cells were analyzed by CytoFLEX flow cytometry (Beckman Coulter, ‎Brea, CA, USA) and analyzed using FlowJo version 10.1 software (Treestar inc, Ashland, OG, USA).

***HLA-C genotyping***

Genomic DNA was isolated from cancer cell lines using QIAamp DNA Mini Kit (Qiagen, Hilden, Germany). PCR was performed using HLA-C SSP PCR Kit (Biosewoom, Korea) according to the manufacturer’s protocol.

***In vivo xenograft tumor model***

Xenograft tumors were established in 7- to 8-week-old female NSG mice (JAbio, Suwon, Republic of Korea). A2780 cells (2 × 10^6^) or A2780cis cells (2 × 10^6^) were subcutaneously inoculated in the right flank, tumor volume was calculated according to the following formula: volume = (width^2^ × length)/2. The animals were randomized into three treatment groups: (1) PBS, (2) cisplatin (2 mg/kg), (3) pNK (1×10^7^). After the tumors reached 90–100 mm3, Cisplatin was intraperitoneally injected to mice once a week for 2 weeks, and pNK cells were intravenously injected twice a week for 2 weeks.

**Table S1. HLA -C genotype of cancer cell lines**

| Cell line | HLA-C Type | | |
| --- | --- | --- | --- |
|  | Our results | Reference | |
| K562 | C*03(C1)/C*05(C2) | C*05,03 | [1] |
| A2780 | C*02(C2)/C*07(C1) | - |  |
| A2780cis | C*02(C2)/C*07(C1) | - |  |
| OVCAR3 | C*07(C1)/C*07(C1) | C*07:02:01,07 | [2] |
| SKOV3 | C*04(C2)/C*05(C2) | C*04,05 | [2] |
| MCF-7 | C*05(C2)/C*05(C2) | C*05:01,05:01 | [1] |
| MCF10A | - | C*07:02,03:03 | [1] |
| MDA-MB-231 | - | C*17:01,02:02 | [1] |
| MDA-MB-468 | - | C*02:02,04:01 | [1] |
| BT20 | - | C*03:03,12:03 | [1] |
| SKBR3 | - | C*03:04',03:04 | [1] |
| T47D | - | C*08:02',08:02 | [1] |

**Table S2. List of used antibodies for FACS**

| Antibodies | Fluorescence | Clone | Cat.No | Supplier |
| --- | --- | --- | --- | --- |
| Antibodies for surface staining | | | | |
| anti-mouse IgG1 kappa isotype | FITC | P3.6.2.8.1 | 11-4714-82 | eBioscience |
| anti-mouse IgG1 kappa isotype | PE | P3.6.2.8.1 | 12-4714-42 | eBioscience |
| anti-mouse IgG1 kappa isotype | PerCP/Cy5.5 | MOPC-21 | 400150 | BioLegend |
| anti-mouse IgG1 kappa isotype | APC | P3.6.2.8.1 | 17-4714-42 | eBioscience |
| anti-mouse IgG1 kappa isotype | APC/Cy7 | MOPC-21 | 400128 | BioLegend |
| anti-mouse IgG1 kappa isotype | eFluor 450 | P3.6.2.8.1 | 48-4714-82 | eBioscience |
| anti-CD3 | eFluor 450 | 48-0038-42 | 48-0038-42 | eBioscience |
| anti-CD3 | FITC | UCHT1 | 11-0038-42 | eBioscience |
| anti-CD56 | PE | CMSSB | 12-0567-42 | eBioscience |
| anti-CD56 | APC | CMSSB | 17-0567-42 | eBioscience |
| anti-CD19 | FITC | HIB19 | 11-0199-42 | eBioscience |
| anti-CD14 | APC/Cy7 | M5E2 | 301820 | BioLegend |
| anti-CD16 | PE | eBioCB16 (CB16) | 12-0168-42 | eBioscience |
| anti-NKG2D | FITC | 1D11 | 11-5878-42 | eBioscience |
| anti-NKp30 | APC | P30-15 | 325210 | BioLegend |
| anti-NKp44 | PE | P44-8 | 325108 | BioLegend |
| anti-NKp46 | PE | 9E2 | 557991 | BD Bioscience |
| anti-OX40 | PE | Ber-ACT35 (ACT35) | 350004 | BioLegend |
| anti-41BB | PE | 4B4 (4B4-1) | 12-1379-42 | eBioscience |
| anti-NKG2C | APC | REA205 | 130-117-398 | Miltenyi Biotec |
| anti-CD94 | PE | DX22 | 12-0949-42 | eBioscience |
| anti-DNAM-1 | PE | 11A8 | 338306 | BioLegend |
| anti-2B4 | APC/Cy7 | C1.7 | 329518 | BioLegend |
| anti-NKG2A | PE | Z199 | IM3291U | Beckman Coulter |
| anti-CD158b | PE | CH-L | 559785 | BD Bioscience |
| anti-CD158e | APC | REA1005 | 130-116-822 | Miltenyi Biotech |
| anti-CD158f | APC-Vio770 | UP-R1 | 130-116-510 | Miltenyi Biotech |
| anti-TIGIT | BB700 | 741182 | 747846 | BD Bioscience |
| anti-BTLA | APC/Cy7 | MIH26 | 344518 | BioLegend |
| anti-CCR2 | PE | K036C2 | 357206 | BioLegend |
| anti-CCR3 | PerCP-Cy™5.5 | 5E8 | 564189 | BD Bioscience |
| anti-CCR4 | APC | REA279 | 130-117-376 | Miltenyi Biotech |
| anti-CCR5 | FITC | HEK/1/85a | MA1-20282 | Invitrogen |
| anti-CCR6 | APC/Cy7 | G034E3 | 353432 | BioLegend |
| anti-CCR7 | FITC | 150503 | 561271 | BD Bioscience |
| anti-TIM-3 | FITC | F38-2E2 | 11-3109-42 | eBioscience |
| anti-LAG-3 | PE | 11C3C65 | 369306 | BioLegend |
| anti-PD-1 | APC | NAT105 | 367406 | BioLegend |
| anti-CD49a | PE | TS2/7 | 328304 | BioLegend |
| anti-CD49b | PE | P1E6-C5 | 359308 | BioLegend |
| anti-Integrin beta 7 | PE | FIB504 | 12-5867-42 | eBiosciences |
| anti-CD62L | APC/Cy7 | DREG-56 | 304814 | BioLegend |
| anti-CD11a | PE | HI111 | 301208 | BioLegend |
| anti-CD18 | FITC | 6.7 | 555923 | BD Bioscience |
| anti-CD2 | FITC | RPA-2.10 | 11-0029-42 | eBiosciences |
| anti-CXCR1 | FITC | eBio8F1-1-4(8F1-1-4) | 11-1819-42 | eBiosciences |
| anti-CXCR2 | PE | eBio5E8-C7-F10(5E8-C7-F10) | 12-1829-42 | eBiosciences |
| anti-CXCR3 | PerCP/Cy5.5 | G025H7 | 353714 | BioLegend |
| anti-CXCR4 | APC | REA649 | 130-109-844 | Miltenyi Biotech |
| anti-CXCR6 | Alexa Fluor® 750 | 56811R | FAB699RS | R&D |
| anti-CD69 | PerCP/Cyanine5.5 | FN50 | 310926 | BioLegend |
| anti-CD57 | APC | REA769 | 130-111-811 | Miltenyi Biotec |
| anti-HLA-G | PE | 87G | 12-9957-41 | eBioscience |
| Antibodies for Intracellular staining | | | | |
| anti-Eomes | FITC | WD1928 | 11-4877-42 | eBiosciences |
| anti-T-bet | PerCP-Cyanine5.5 | eBio4B10 (4B10) | 45-5825-82 | eBiosciences |
| anti-Ki-67 | PE | Ki-67 | 350504 | BioLegend |
| anti-GATA-3 | APC | REA174 | 130-100-649 | Miltenyi Biotech |
| anti-E4BP4 | Alexa Fluor® 750 | 714401 | FAB8570S | R&D |

**Table S3.** **List of used antibodies for blocking assay**

| Antibodies | Working con. | Clone | Cat.No | Supplier |
| --- | --- | --- | --- | --- |
| anti-mouse IgG1k | 1µg/100µl | MOPC-21 | 400124 | BioLegend |
| anti-NKG2D | 2µg/100µl | 149810 | MAB139 | R&D |
| anti-NKp30 | 2µg/100µl | P30-15 | 325204 | BioLegend |
| anti-NKp44 | 1µg/100µl | P44-8 | 325104 | BioLegend |
| anti-NKp46 | 2µg/100µl | 9E2 | 331904 | BioLegend |
| anti-DNAM1 | 1µg/100µl | DX11 | 559786 | BD Bioscience |
| anti-KIR2DL1 | 2.5µg/100µl | 143211 | MAB1844 | BD Bioscience |
| anti-KIR2DL3 | 2.5µg/100µl | 180701 | MAB2014 | BD Bioscience |
| anti-KIR3DL1 | 2.5µg/100µl | DX9 | MAB1225 | BD Bioscience |

**Table S4.** **Primer sequences for qRT-PCR**

| **Gene** | **Primers** |
| --- | --- |
| *HLA-C1* | Forward: CGCCGCGAGTCCGAGAGG  Reverse: GTTGTAGTAGCCGCGCAGG |
| *HLA-C2* | Forward: CGCCGCGAGTCCGAGAGG  Reverse: GTTGTAGTAGCCGCGCAG |
| *HLA-C* | Forward: GGAGACACAGAAGTACAAGCGC  Reverse: ACATCCTCTGGAGGGTGTGAGA |
| *GAPDH* | Forward: ACCACAGTCCATGCCATCAC  Reverse: TCCACCACCCTGTTGCTGTA |

**Table S5.** **Summary of NK culture conditions and characteristics**

| **Cell Source** | **Culture condition** | **CD3^-^CD56^+^ purity (% at Day14)** | **Expansion (fold change at D14)** | **Expression of activating receptors (% of Day14 NK cells)** | **Ref** |
| --- | --- | --- | --- | --- | --- |
| Sorted CD3^-^CD56^+^ cells from PBMCs | ● γ-globulin and anti-NKp46-coated flask  ● Alys505NK medium with 1000 IU/mL IL-2, 50 ng/mL IL-18, and 5% heat-inactivated autologous plasma | 99.7% ± 0.2 | 175±43 | CD16: 94.88 ± 2.11  NKG2D: 93.63±9.44  NKp30: 85.38±11.89  NKp44: 91.28 ±5.2  NKp46: 78.03±11.19  *CCR5: 88.15± 3.21 | This study |
| CD3^+^ T cell  -depleted PBMCs | ● KBM501 medium with 2813 IU/ mL IL-2, 2000 mg/L human serum albumin, and 60 mg/L kanamycin sulfate | 96 | ~200 | CD16: >90  NKG2D: >90  NKp30: >90  NKp44: no data  NKp46: >80 | [3] |
| CD3^+^ T cell  -depleted PBMCs | ● Irradiated autologous PBMCs  ● CellGro SCGM medium with 1% auto-plasma, 10 ng/mL anti-CD3 monoclonal antibody OKT3 and 500 IU/mL IL-2 | 98.10±0.88 | 691.4±170.2 | CD16: no data  NKG2D: 80-99  NKp30: 45-90  NKp44: 30-80  NKp46: 30-90  * CCR5: <5 | [4, 5] |
| Non-NK cell depleted PBMCs | ● Anti-human CD16-coated culture plates.  ● Irradiated autologous PBMCs  ● Lymphocyte Growth medium 3 with 500 IU/ml IL-2 and 5% of human serum | >98% | 794 ± 115.6 | CD16:90  NKG2D: 100  NKp30: 80  NKp44: 90  NKp46: 80 | [6] |
| NK cell enriched lymphocytes from PBMCs | ● Irradiated K562-mbIL15-41BBL cells  ● 200 IU/ml human IL-2 in cRPMI | 45.6 ± 27.4 | 166.3 ± 211.5 | CD16: no data  NKG2D:96 (93-99)  NKp30: 82 (67-97)  NKp44: 59 (16-93)  NKp46: 87 (64-97) | [7] |
| Sorted CD3^-^CD56^+^ cells from PBMCs | ● CellGenix® GMP Stem Cell Growth medium with 10% human serum, 10 ng/ml IL-2, 10 ng/ml IL-15, IL-27 and 10 ng/ml IL-18 | 93.1~97.5 | 17.19 ± 4.85 | CD16: 86.76 ± 10.43  NKG2D: 95.64± 5.68  NKp30: 80.09± 18.93  NKp44: 28.85 ± 15.09  NKp46: 28.38± 23.38 | [8] |

**Table S6. Expansion of NK cells with our culture method from different cell sources**

|  |  | PBMCs | CD3+ depleted PBMCs | CD3-CD56+ cells from PBMCs |
| --- | --- | --- | --- | --- |
| D0 | NK cells (%) | 11 | 20 | 99 |
|  | Total cell numbers | 2x10^7^ | 2x10^7^ | 2x10^7^ |
|  | NK cell numbers | 2.2x10^6^ | 4x10^6^ | 2x10^7^ |
| D14 | NK cells (%) | 85 | 99 | 99 |
|  | Total cell numbers | 4x10^9^ | 4x10^9^ | 4x10^9^ |
|  | NK cell numbers | 3.4x10^9^ | 4x10^9^ | 4x10^9^ |
| Fold expansion of NK cells | | 1545 | 1000 | 200 |

***Supplementary Figure & Figure Legends***


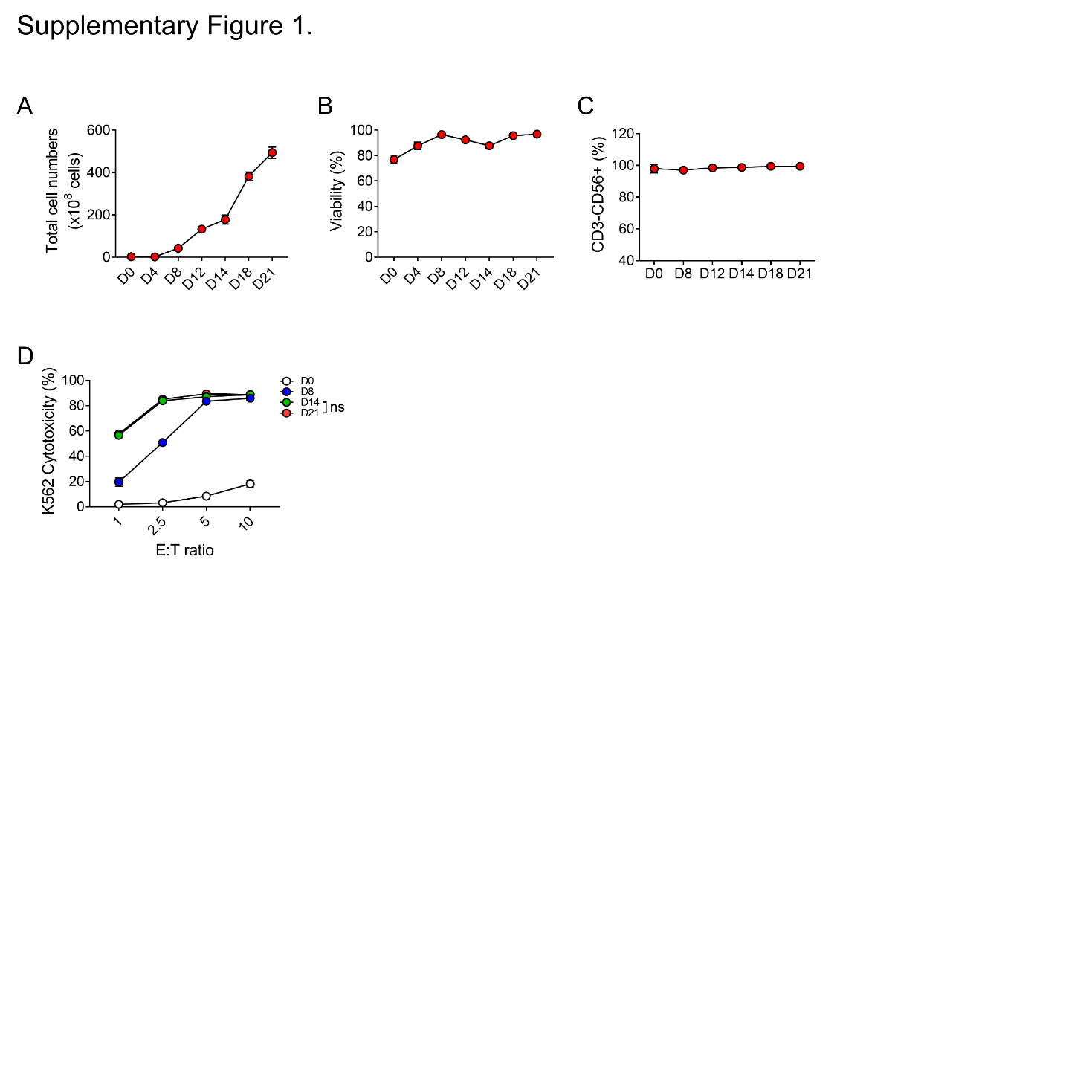


**Figure S1. Characterization of *ex vivo-*expanded pNK cells for 21 days.**

(A) Total cell number of pNK cells was assessed after 21 days of culture (n=3). (B) The viability of expanded pNK cells at indicated time point was evaluated by staining of propidium iodide. (C) Purity of NK cells at indicated time point (n=3). (D) Cytotoxicity of pNK cells against K562 at the indicated E:T ratio was compared before (D0) and after (D8, D14, D21) pNK cell expansion (n=3). Statistical analysis was performed using two-way ANOVA (ns; non-significant, D14 vs D21). The data represented as mean ± SD.


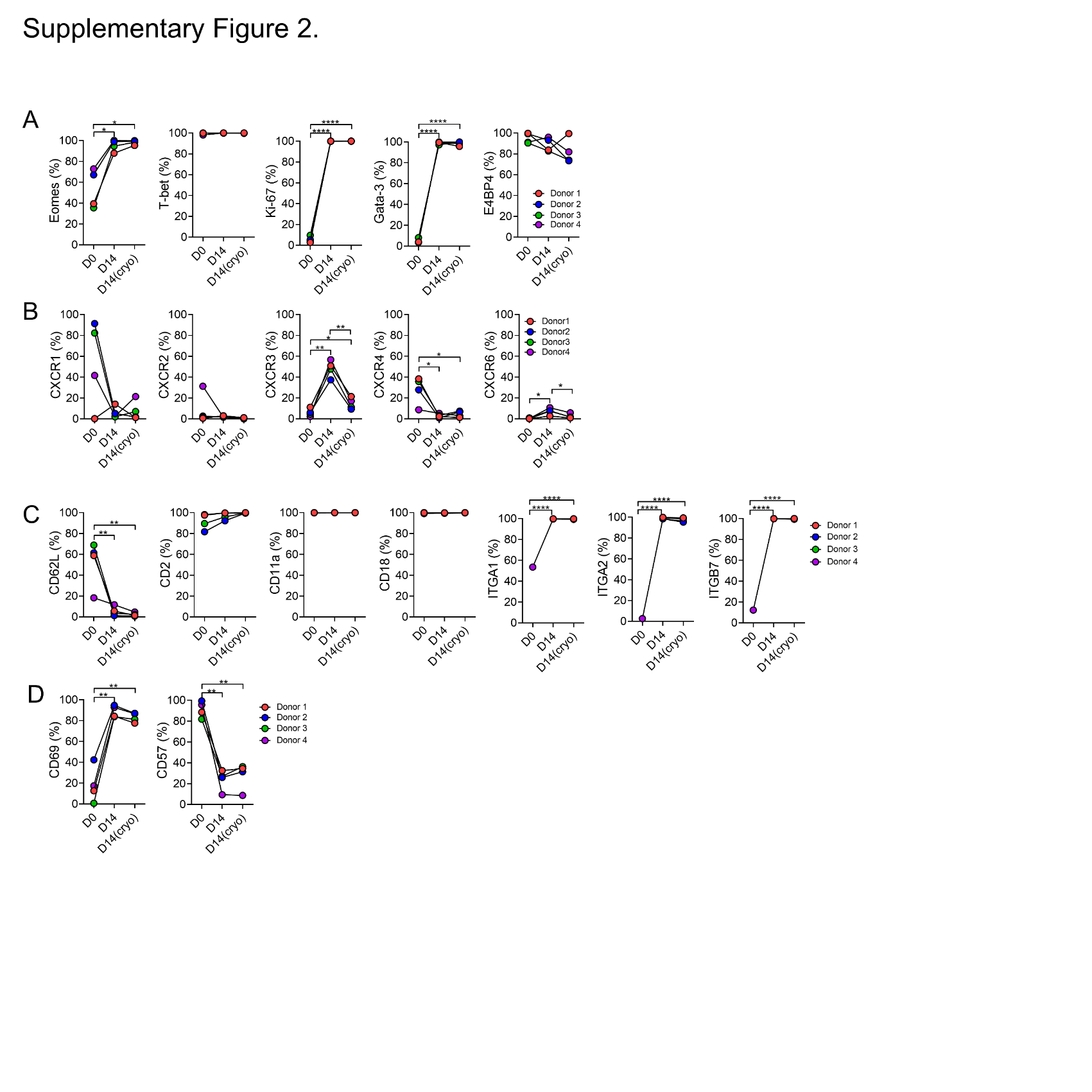


**Figure S2. Flow cytometry analysis of pNK cells for transcription factors, chemokine receptors, adhesion molecules, and maturation markers before expansion, after expansion, and after cryopreservation.**

Surface expression of transcription factors (A), chemokine receptors (B), adhesion molecules (C), and maturation markers (D) was analyzed by flow cytometry before (D0) and after expansion (D14) and after cryopreservation (D14 cryo) of pNK cells from 4 different donors. Statistical analysis was performed using a paired t-test (**P* < 0.05, ***P* < 0.01, ****P* < 0.001 and *****P* < 0.0001. D0 was compared with D14 or D14 cryo. Furthermore, D14 was compared with D14 cryo.


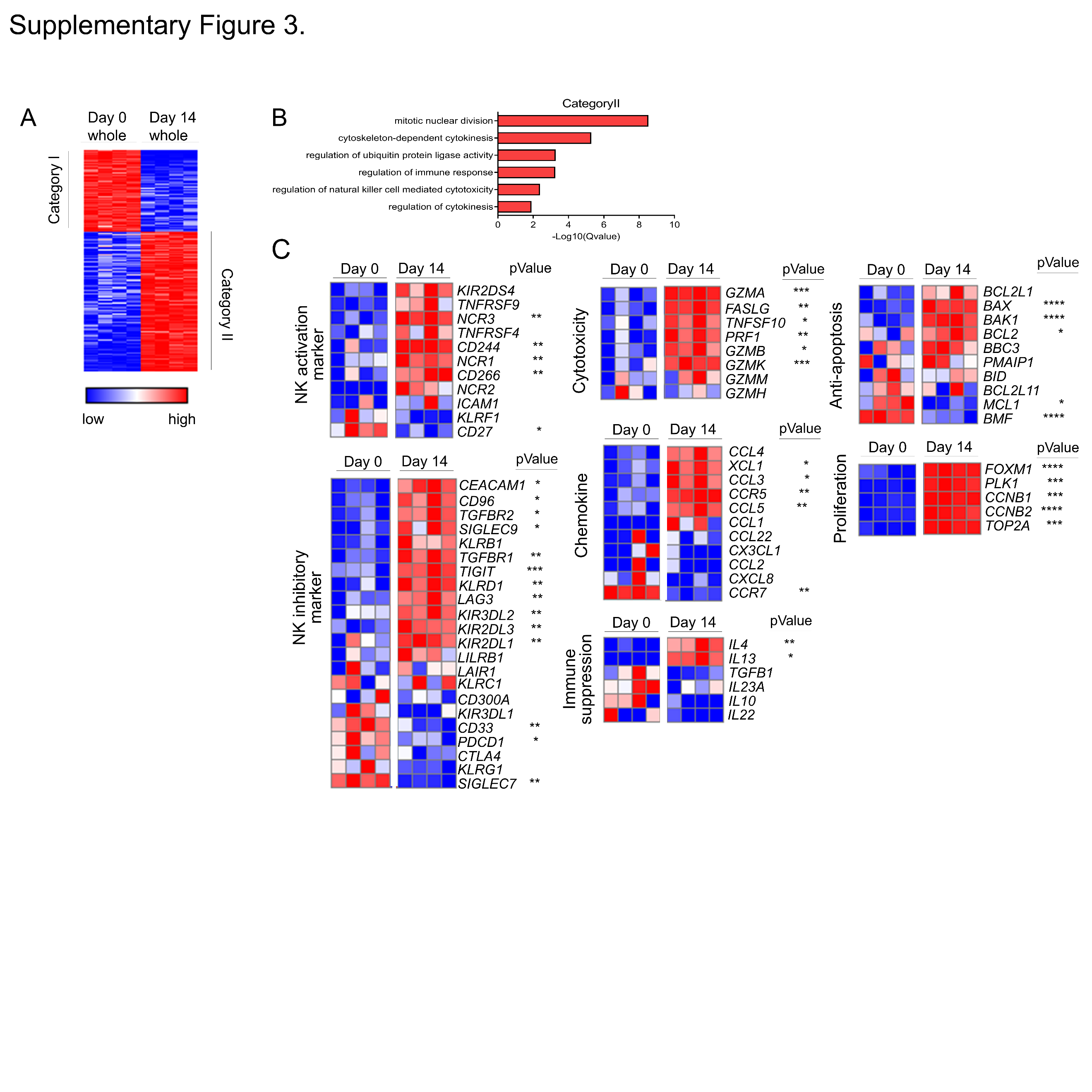


**Figure S3. Gene expression signature of cryopreserved pNK cells from leukapheresis products without CD3^-^CD56^+^ selection**

(A-C) Genome-wide transcriptional profiles of before (Day 0) and after (Day 14) pNK cell expansion. (A) upregulated gene set at Day 0 (Category I) and Day14 (Category II). (B) Gene Ontology Biological Process (GOBP) analysis of up-regulated genes at Day 14. (C) Heat maps representing genes (labels to the right of plots) clustered in GOBP NK activation, NK inhibition, cytotoxicity, chemokine, immune suppression, anti-apoptosis and proliferation (labels to the left of plots) with scaled intensities. Warm color (red) denotes an increase in gene expression whereas cold color (blue) indicates a decrease as compared to the average level of gene expression in day0 pNK cells.

**
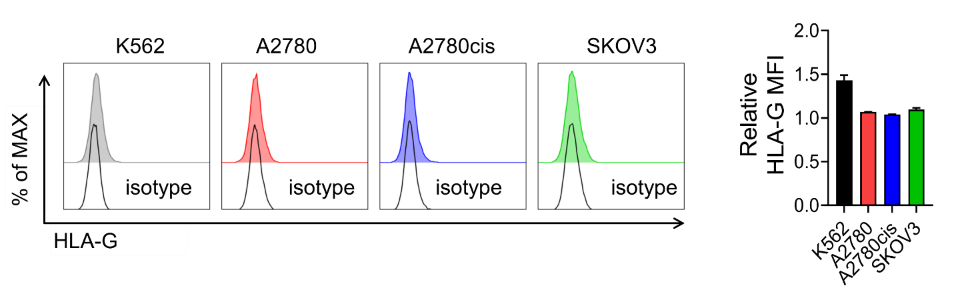
**

**Figure S4. Expression of HLA-G in three different ovarian cancer cell lines.** Expression of HLA-G in K562 and three different ovarian cancer cell lines was analyzed using flow cytometry. Left: representative histograms of the expression in each cell lines. Right: mean fluorescence intensity determined using flow cytometry. Relative HLA-G MFI was calculated by each isotype control.


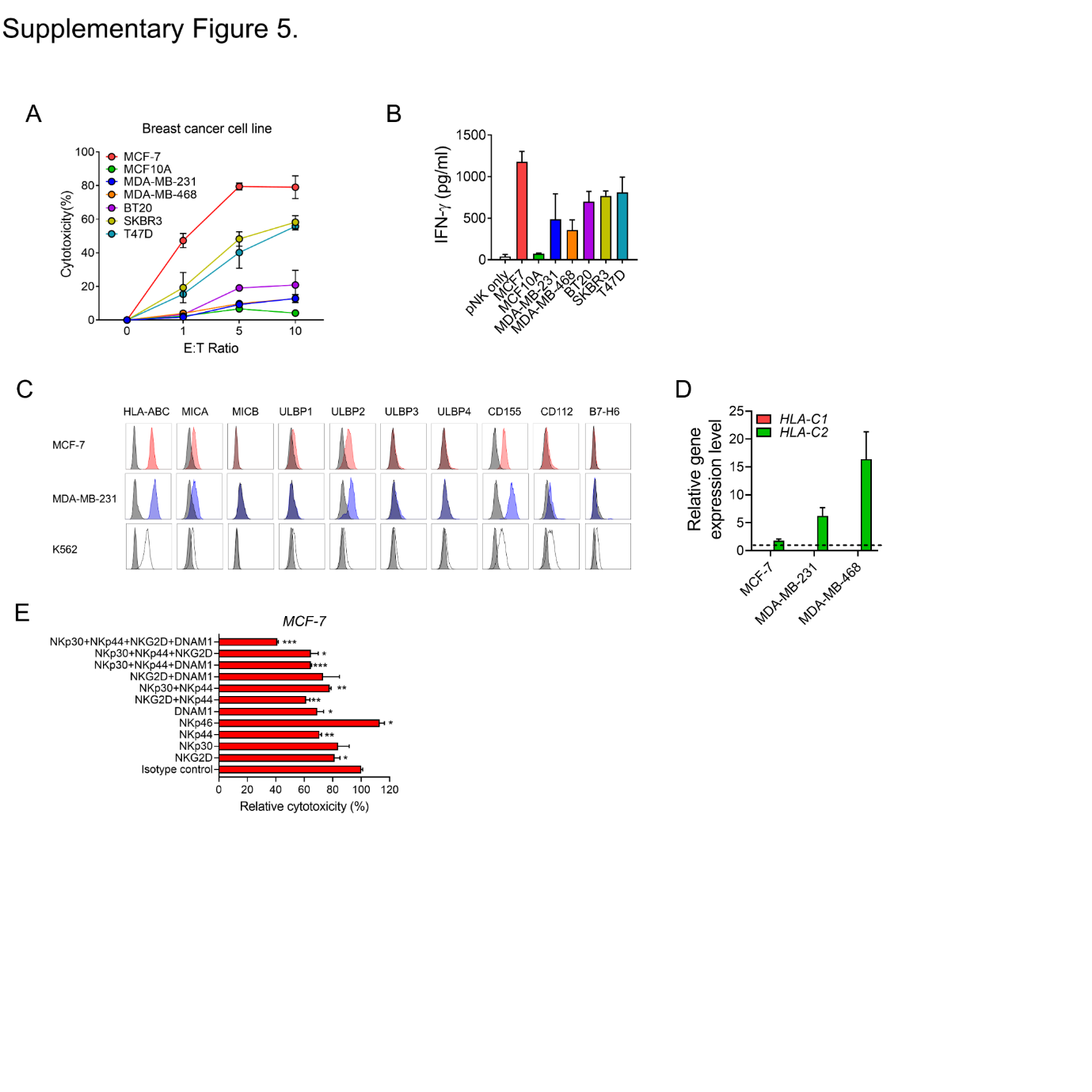


**Figure S5. Cytotoxicity of cryopreserved pNK cells against breast cancer cell lines.**

(A) Cytotoxicity of cryopreserved pNK cells against various breast cancer cell lines was analyzed using CFSE/7-AAD assay with the indicated Effector:Target (E:T) ratio. The data represented as mean ± SD. (B) cryopreserved pNK cells were cultured with various breast cancer cell lines for 4h at E: T = 1:10 ratio. Interferon-γ (IFN-γ) levels in the supernatant were detected by ELISA. The data represented as mean ± SD. (C) Expression of HLA-class I, ULBP-1, ULBP-2, MIC-A/B, CD112 and CD155 in MCF-7 and MDA-MB-231 was analyzed using flow cytometry. Representative histograms of the expression of receptors in each cell lines. Grey histograms represent isotype controls. (D) Expression of HLA-C1 and HLA-C2 genes in 5 cancer cell lines. The data represented as mean ± SD. (E) Cryopreserved pNK cells were pre-incubated with blocking antibodies against NKG2D, NKp30, NKp44 and DNAM-1 and cytotoxicity was analyzed against MCF-7 cells using CFSE/7-AAD assay in triplicate. E:T ratio was 10:1. Percent inhibition of cytotoxicity was calculated as the percentage of the inhibition via the isotype control antibody. Date are represented as mean ± SD. Each group was compared to the isotype control. Statistical analysis was performed using an unpaired *t*-test (**P*<0.05, ***P*<0.01 and ****P*<0.001).


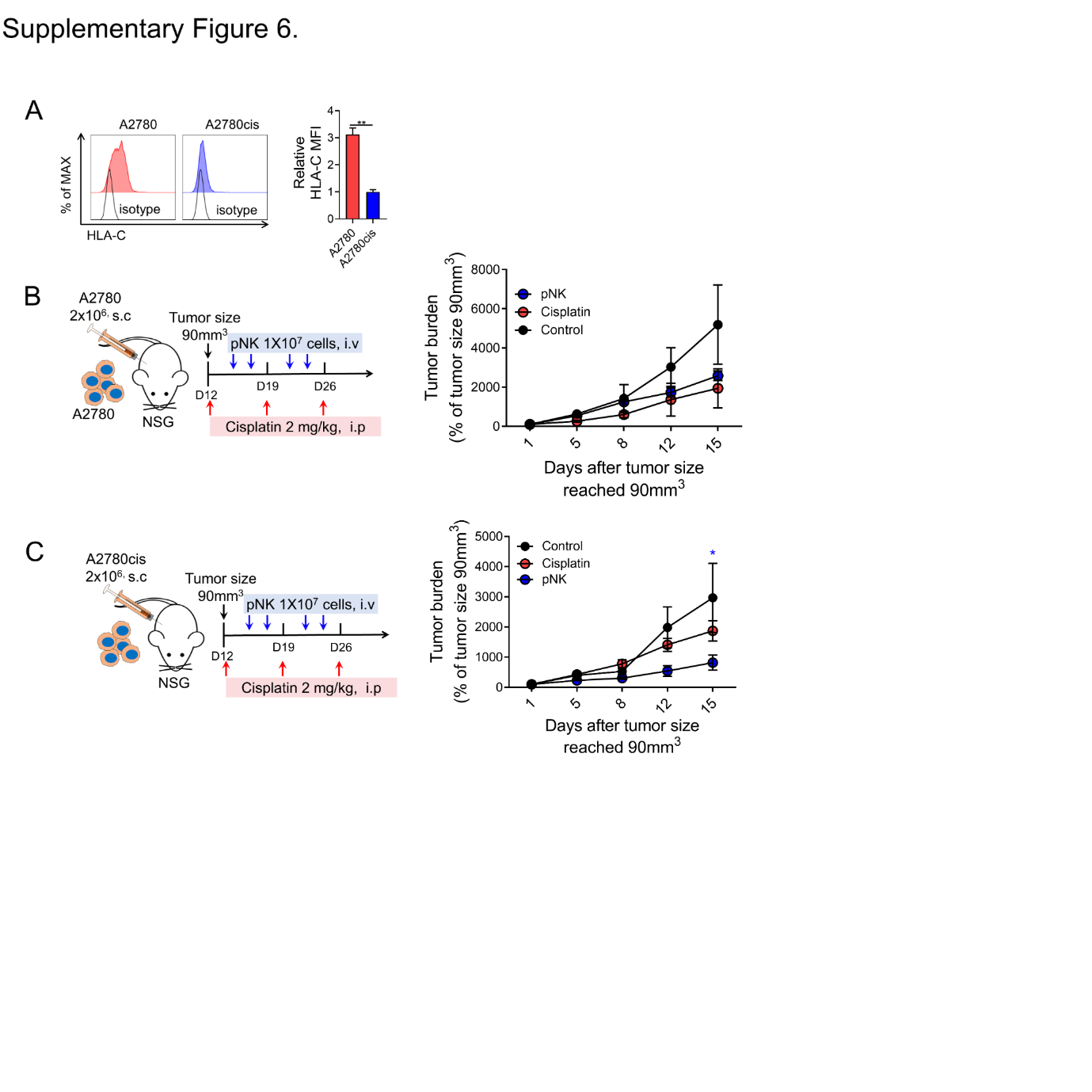


**Figure S6. *In vivo* therapeutic effects of cryopreserved pNK cells against A2780 or A2780cis xenograft tumors. (a)** Expression of HLA-C in A2780 and A2780cis was analyzed using flow cytometry. Left: representative histograms of the expression in each cell line. Right: mean fluorescence intensity determined using flow cytometry. The relative HLA-C MFI was calculated using the level of each isotype control. Data are represented as mean±SD. Statistical analysis was performed using an unpaired t-test (***P<* 0.01). A2780 cells (2x10^6^) **(b)** or A2780cis (2x10^6^) **(c)** were subcutaneously inoculated into the right flank and tumors were allowed to grow to a size of 90–100 mm^3^ prior to initiation of experiments. Cisplatin (2 mg/kg) was intraperitoneally injected into the mice once a week (red arrow), and 1x10^7^ pNK cells were intravenously injected into the mice twice a week (blue arrow) (n = 3 per group). Tumor volume was measured on the indicated days after tumor injection. Statistical analysis was performed using two-way ANOVA (**P <* 0.05 as compared to the control group).


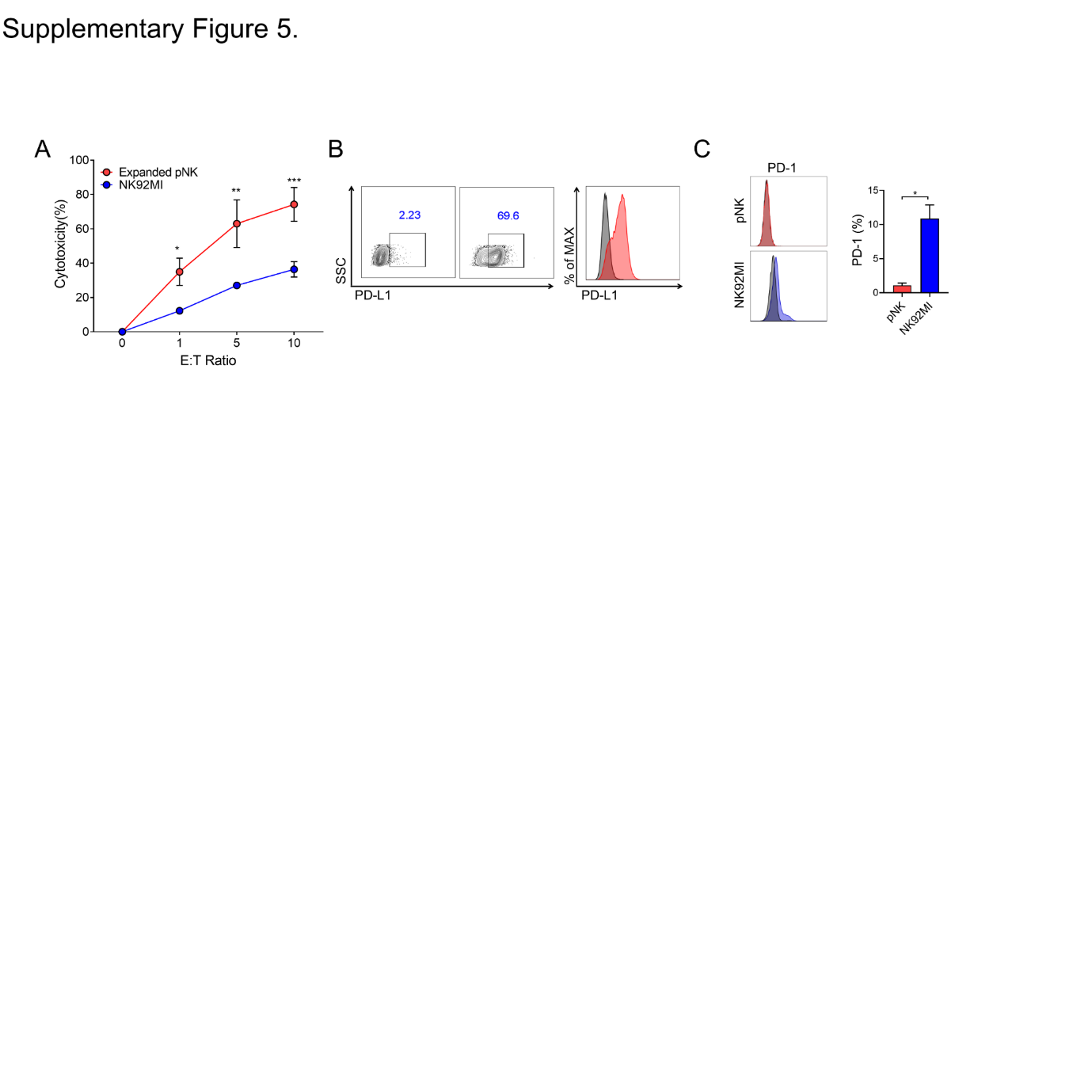


**Figure S7. Effect of PD-1/PD-L1 axis on pNK cytotoxicity against A2780cis**

(A) Cytotoxicity of cryopreserved pNK cells and NK92MI cells against A2780cis cells was analyzed using CFSE/7-AAD assay with the indicated Effector:Target (E:T) ratio. The data represented as mean ± SD Statistical analysis was performed by means of two-way anova (**P* < 0.05, ***P* < 0.01 and ****P* < 0.001) (B) Flow cytometry analysis of PD-L1 expression of A2780cis cells. Representative histogram plot is shown in the right panel. (C) Flow cytometry analysis of PD-1 expression of pNK and NK92MI cells. Grey histograms represent isotype controls. Mean fluorescence intensity is shown in the right panel. The data represented as mean ± SD Statistical analysis was performed by performed using an unpaired t-test (**P* < 0.05).

***References***

1. Boegel, S., et al., *A catalog of HLA type, HLA expression, and neo-epitope candidates in human cancer cell lines.* Oncoimmunology, 2014. **3**(8): p. e954893.

2. Adams, S., et al., *HLA class I and II genotype of the NCI-60 cell lines.* J Transl Med, 2005. **3**(1): p. 11.

3. Saito, S., et al., *Ex vivo generation of highly purified and activated natural killer cells from human peripheral blood.* Hum Gene Ther Methods, 2013. **24**(4): p. 241-52.

4. Lim, O., et al., *GMP-compliant, large-scale expanded allogeneic natural killer cells have potent cytolytic activity against cancer cells in vitro and in vivo.* PLoS One, 2013. **8**(1): p. e53611.

5. Min, B., et al., *Optimization of Large-Scale Expansion and Cryopreservation of Human Natural Killer Cells for Anti-Tumor Therapy.* Immune Netw, 2018. **18**(4): p. e31.

6. Lee, H.R., et al., *Expansion of cytotoxic natural killer cells using irradiated autologous peripheral blood mononuclear cells and anti-CD16 antibody.* Sci Rep, 2017. **7**(1): p. 11075.

7. Voskens, C.J., et al., *Ex-vivo expanded human NK cells express activating receptors that mediate cytotoxicity of allogeneic and autologous cancer cell lines by direct recognition and antibody directed cellular cytotoxicity.* J Exp Clin Cancer Res, 2010. **29**: p. 134.

8. Choi, Y.H., et al., *IL-27 enhances IL-15/IL-18-mediated activation of human natural killer cells.* J Immunother Cancer, 2019. **7**(1): p. 168.
